# Plasma TNF-α and Soluble TNF Receptor Levels after Doxorubicin with or without Co-Administration of Mesna—A Randomized, Cross-Over Clinical Study

**DOI:** 10.1371/journal.pone.0124988

**Published:** 2015-04-24

**Authors:** John Hayslip, Emily V. Dressler, Heidi Weiss, Tammy J. Taylor, Mara Chambers, Teresa Noel, Sumitra Miriyala, Jeriel T. R. Keeney, Xiaojia Ren, Rukhsana Sultana, Mary Vore, D. Allan Butterfield, Daret St Clair, Jeffrey A. Moscow

**Affiliations:** 1 University of Kentucky, Markey Cancer Center, Lexington, Kentucky, United States of America; 2 University of Kentucky, Division of Hematology and Blood and Marrow Transplantation, Lexington, Kentucky, United States of America; 3 University of Kentucky, Department of Pediatrics, Lexington, Kentucky, United States of America; 4 University of Kentucky, Division of Medical Oncology, Lexington, Kentucky, United States of America; 5 University of Kentucky, Graduate Center for Toxicology, Lexington, Kentucky, United States of America; 6 University of Kentucky, Department of Chemistry, Lexington, Kentucky, United States of America; Colorado State University, UNITED STATES

## Abstract

**Purpose:**

Chemotherapy-induced cognitive impairment (CICI) is a common sequelae of cancer therapy. Recent preclinical observations have suggested that CICI can be mediated by chemotherapy-induced plasma protein oxidation, which triggers TNF-α mediated CNS damage. This study evaluated sodium-2-mercaptoethane sulfonate (Mesna) co-administration with doxorubicin to reduce doxorubicin-induced plasma protein oxidation and resultant cascade of TNF-α, soluble TNF receptor levels and related cytokines.

**Methods:**

Thirty-two evaluable patients were randomized using a crossover design to receive mesna or saline in either the first or second cycle of doxorubicin in the context of a standard chemotherapy regimen for either non-Hodgkin lymphoma or breast cancer. Mesna (360 mg/m^2^) or saline administration occurred 15 minutes prior and three hours post doxorubicin. Pre-treatment and post-treatment measurements of oxidative stress, TNF-α and related cytokines were evaluated during the two experimental cycles of chemotherapy.

**Results:**

Co-administration of mesna with chemotherapy reduced post-treatment levels of TNF-related cytokines and TNF-receptor 1 (TNFR1) and TNF-receptor 2 (TNFR2) (p = 0.05 and p = 0.002, respectively). Patients with the highest pre-treatment levels of each cytokine and its receptors were the most likely to benefit from mesna co-administration.

**Conclusions:**

The extracellular anti-oxidant mesna, when co-administered during a single cycle of doxorubicin, reduced levels of TNF-α and its receptors after that cycle of therapy, demonstrating for the first time a clinical interaction between mesna and doxorubicin, drugs often coincidentally co-administered in multi-agent regimens. These findings support further investigation to determine whether rationally-timed mesna co-administration with redox active chemotherapy may prevent or reduce the cascade of events that lead to CICI.

**Trial Registration:**

clinicaltrials.gov NCT01205503.

## Introduction

Symptoms of chemotherapy-induced cognitive impairment (CICI), or ‘chemobrain’, are common complaints of cancer survivors. While incidence estimates of CICI vary widely [1], CICI is recognized as a significant morbidity by the National Cancer Institute and patient advocacy groups, and therefore is a high priority in survivorship research. There is a lack of consensus regarding the causality of CICI [2], and this lack of mechanistic understanding has hampered efforts to prevent CICI. Our working model proposes that oxidative stress and increased systemic inflammatory cytokines leads to CNS damage and subsequent CICI symptoms in cancer survivors. Our group has proposed a mechanism for CICI, in which a TNF-α-mediated process is initiated by the direct intravascular oxidative modification of plasma proteins by chemotherapy, followed by monocyte release of TNF-α, with tissue damage then resulting as a downstream effect of TNF-α [3]. This systemic TNF-α crosses the blood-brain barrier, activates latent microglia producing yet more TNF-α within the brain, and promulgates a cascade of events that results in cognitive symptoms. Evidence to support this model includes our prior demonstration that systemic Doxorubicin (Dox) administration in mice results in increased brain oxidative stress, even though neither Dox nor its major metabolite has the ability to cross the blood brain barrier and enter the CNS [4]; that anti-TNF-α antibody prevents the Dox-mediated CNS injury in animal models, demonstrating that systemic TNF-α plays a role in Dox-mediated brain injury [5]; that brain mitochondrial respiration decreases after TNF-α mediated nitric oxide induction, demonstrating that TNF-α can directly cause the same type of CNS injury that is observed after Dox administration [6]; and that TNF-α activates brain microglia to produce even more TNF-α in the microenvironment, demonstrating how TNF-α can stimulate a positive feedback loop to amplify the damage initiated by Dox [7].

Therefore, preventing chemotherapy-induced plasma protein oxidation without hindering tumor intracellular oxidative stress may prevent the initiation of this cascade and thereby prevent CICI without reducing the efficacy of the chemotherapy. In contrast to other antioxidants, such as N-acetyl-cysteine, sodium-2-mercaptoethane sulfonate (mesna) is a drug that was specifically found to have poor cellular uptake by most cell types [8]. This specificity allows mesna to act as an antioxidant only in blood and urine, without interfering with the anticancer activity of antineoplastic chemotherapy. To support the potential of mesna to block our proposed mechanism of CICI, we have shown that mesna co-administration blocks Dox-mediated plasma APOA1 oxidation and reduces TNF-α generation in a macrophage cell line [3]; that mesna directly interferes with Dox-mediated DCF fluorescence, demonstrating mesna-mediating scavenging of Dox-mediated ROS [3], and that *in vivo* mesna co-administration with Dox prevents Dox-induced plasma oxidative stress in mice [9].

We hypothesized that rationally-timed administration of mesna with Dox-containing chemotherapy could reduce patient plasma protein oxidation and plasma inflammatory cytokine levels as compared to patients receiving Dox-containing chemotherapy alone. Consequently, we conducted a pilot study in which cancer patients undergoing therapy with regimens that included Dox received their first two prescribed doses of Dox with or without mesna in a randomized order, and then went on to receive the remainder of therapy in a standard fashion. The primary outcomes were measures of plasma TNF-α, TNF plasma receptor levels which typically increase in response to TNF-α signaling, IL-18 a downstream messenger of TNF-α and inflammatory signaling, and plasma protein oxidation.

## Methods

### Objectives

The primary objective was to determine if co-treatment with mesna significantly reduces plasma protein oxidation and plasma TNF-α levels in patients receiving Dox-containing chemotherapy. The secondary objective was to determine if mesna prevented biochemical evidence of cardiac myocyte injury after Dox administration. The University of Kentucky’s Medical Institutional Review Board reviewed and approved this clinical study prior to engaging any participants and all participants were enrolled from the University of Kentucky’s Markey Cancer Center. This study was listed with clinicaltrials.gov (NCT01205503). All involved participants provided written informed consent.

### Patients

**Fig 1:**
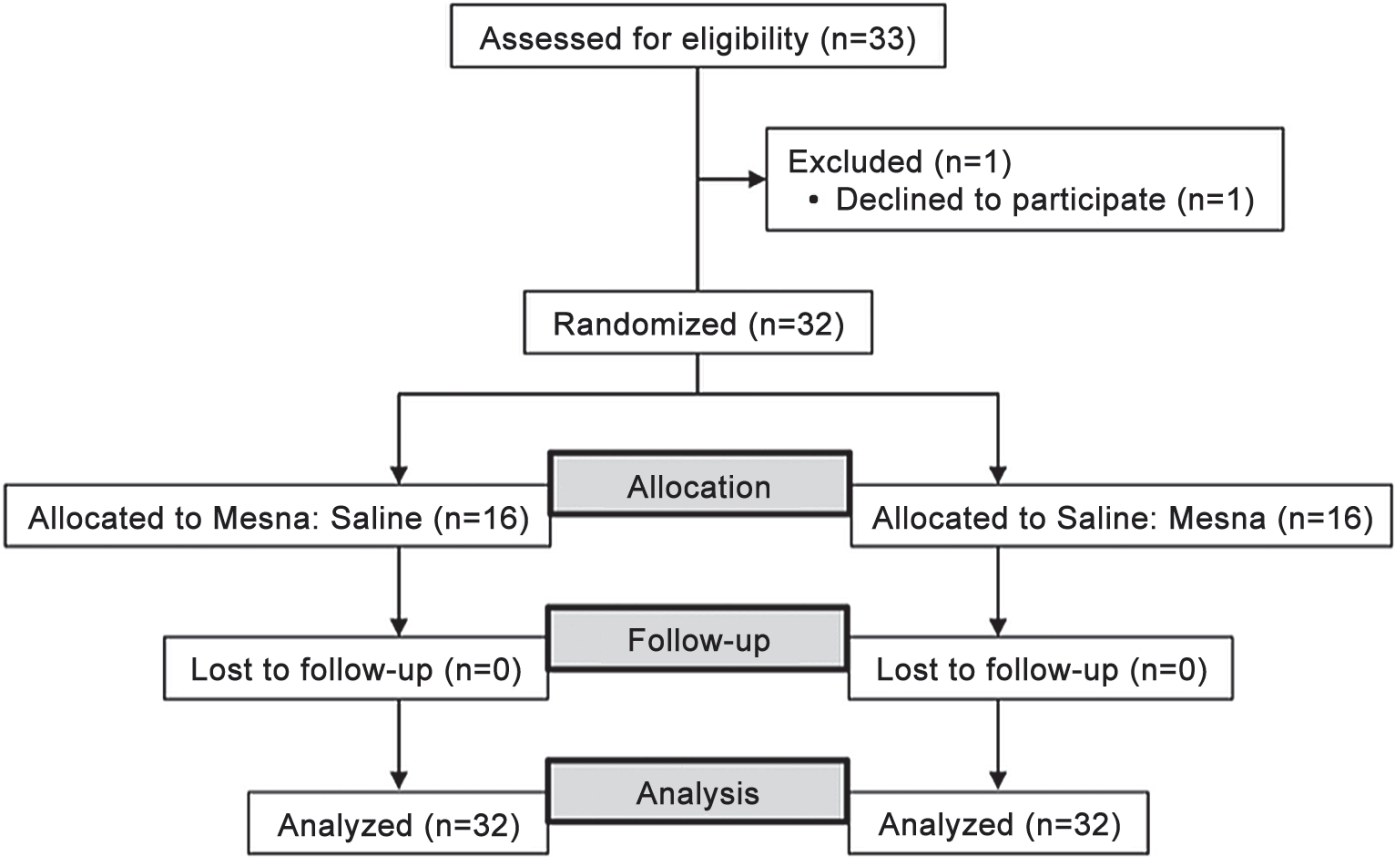
Flow of patient enrollment.

Eligible participants were required to have histologically or cytologically confirmed breast cancer or non-Hodgkin lymphoma that required one of the following treatment regimens as their standard of care treatment: Dox 60 mg/m2 and cyclophosphamide 600 mg/m2 (AC); Dox 50 mg/m2, cyclophosphamide 500 mg/m2 and docetaxel 75 mg/m2 (TAC); or Dox 50 mg/m2, cyclophosphamide 750 mg/m2, vincristine 1.4 mg/m2 (capped at 2 mg dose) and prednisone 100 mg +/- rituximab 375 mg/m2 (CHOP+/-R). Additional inclusion criteria included age >18 years with normal hematopoietic, hepatic and renal functions. A projected sample size of 32 evaluable participants was planned with enrollment of up to an additional eight participants in the event of nonevaluable participants. Modeling from protein-bound 4-hydroxyenol reductions with mesna co-administration as compared to Dox alone in a previous observational study [3], 32 evaluable subjects afford 80% power at α = 0.05 supposing that the observed mesna effect is 50% of what was observed in the previous study. A total of 33 participants were enrolled and 32 were eligible for analysis (Fig 1).

### Study Design

**Fig 2:**
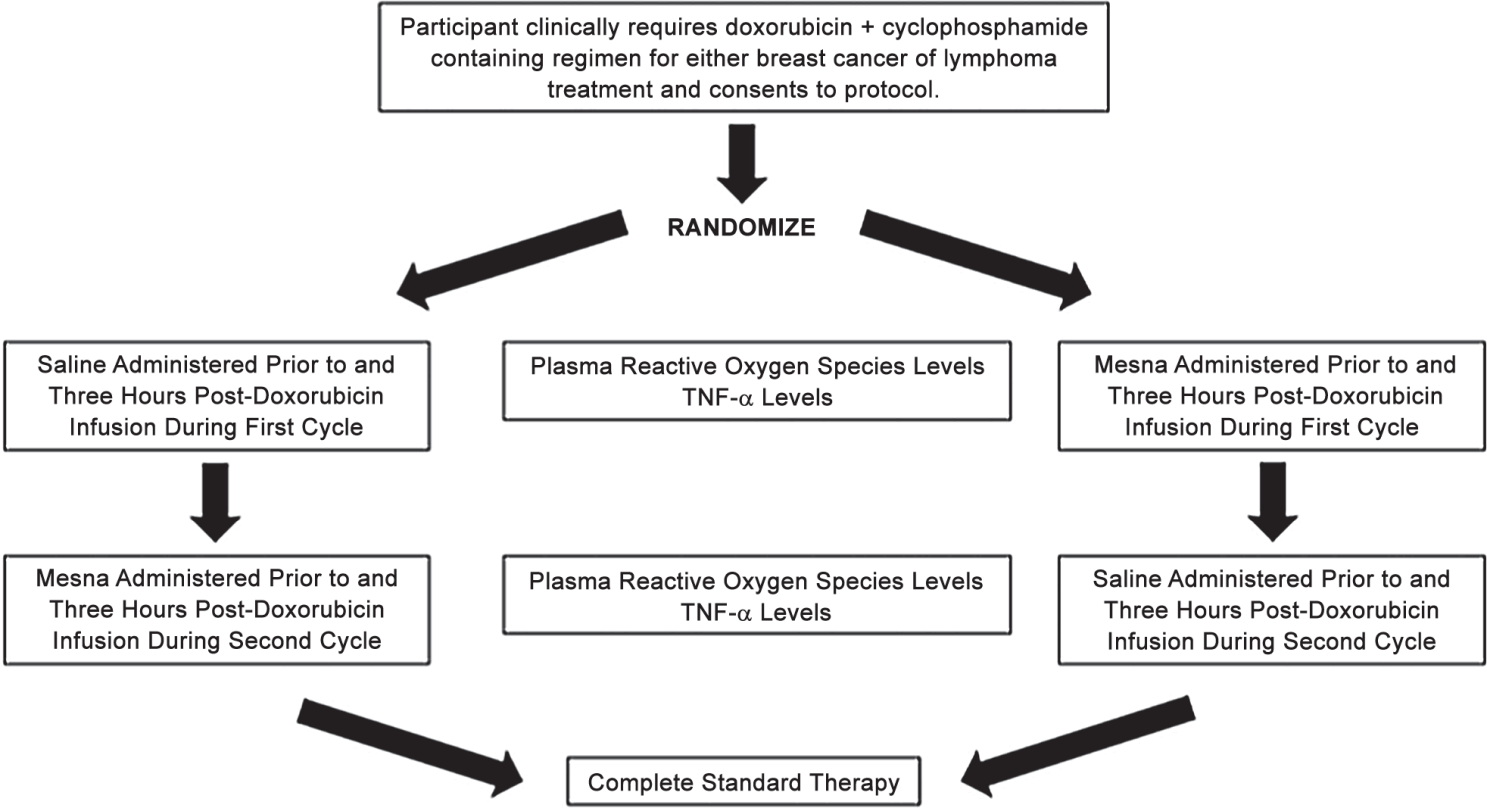
Study design.

The study design is shown in Fig 2. Each participant received one cycle of Dox with mesna 15 minutes prior to administration and three hours post administration, and another cycle with saline instead of mesna. Mesna was administered twice because of mesna’s short plasma half-life of 1.17 hours [10] to optimize the protective effect throughout Dox’s multiphasic plasma clearance during which 90% or greater of doxorubicin and metabolites are cleared within six hours of administration [11]. Patients were randomized in a crossover design to receive mesna versus saline on the first or second cycle because it was not known if the order would affect the outcome variables. Stratified block randomization with stratification for tumor type between lymphoma and breast cancer was used with an 1:1 allocation ratio. The sequential randomization scheme was determined by the study statistician based off a SAS macro for blocked randomization with varying block sizes of 4 and 6 [12] and all other investigators where blinded to which cycles contained mesna and which contained saline. Upon enrollment, each new participant was given a participant number. Only the pertinent pharmacist and statistician had access to the randomization key. All participants were followed clinically during these cycles of therapy and adverse events were assessed and classified using the Common Terminology Criteria for Adverse Events version 4. During the experimental cycles, blinded mesna (360 mg/m2) or saline was infused over 15 minutes, then Dox infused over 15 minutes and then a repeat dose of assigned mesna or saline was given three hours after the Dox began. To isolate the treatment effect of mesna co-administration with Dox, the remainder of the chemotherapy regimen was held until the six-hour post treatment serum samples were collected for analyses and then the remainder of the prescribed regimen was infused as per standard practice. After these two experimental cycles, all participants received the remainder of their clinically indicated chemotherapy off study as per standard of care.

### Statistical Analysis

Descriptive statistics were calculated to summarize each of the cytokine and biochemical measures at each time point of follow-up for each treatment group. Baseline characteristics were compared between groups using Fisher’s exact tests or two-sample t-tests where appropriate. Normality assumptions were checked using qqplots and all measures were log transformed to better fit with parametric modeling; thus all summary statistics are presented with geometric means and 95% confidence intervals for the back transformed data (S1 Table) [13]. All crossover analyses were conducted in SAS 9.3 using proc mixed. However, there were significant carry-over effects for the majority of measures. Based on the cross-over model results, a repeated measures model was performed using the three time points prior to the crossover [14]. All initial models were adjusted for baseline biochemical measure levels, time of measurement, treatment received (mesna or saline), treatment type (AC, TAC, CHOP) as the study design was stratified randomized on this criteria and corresponding first-order interactions. Best models were selected using backward selection and AIC criterion. All analyses presented were intent to treat. Additional analyses included calculation of Spearman Correlation coefficients between baseline biochemical measures and descriptive summaries of adverse event data.

### Plasma Samples

During cycles one and two of each participant’s planned treatment, blood samples were obtained immediately prior to test-article administration and six hours after the infusion of Dox. The blood samples were placed on ice at the bedside and the plasma was prepared within 30 minutes of being drawn to ensure as little modification of plasma proteins as possible. Once the plasma samples were prepared, they were snap frozen and stored at -80°C until analyses.

### Methods for laboratory studies

Oxidative Stress Assays: The slot-blot method was used to determine levels of protein oxidation [indexed by protein carbonyl (PC) and 3-nitrotyrosine (3-NT)] and lipid peroxidation [indexed by protein-bound 4-hydroxy-2-nonenal (HNE) as previously described [3, 9].

Enzyme-linked Immunosorbent assay (ELISA): Plasma samples were used to measure TNF, TNFR1, TNFR2 and IL-18 levels, according to the human ELISA following the manufacturer’s instructions (R&D Systems, Minneapolis, MN). The TNF, TNFR1, TNFR2 and IL-18 concentration in the sample was calculated from the recombinant human TNF and its receptors as well as IL-18 standard curves. The means of minimum detectable dose of the assays were 0.106 pg/ml, 0.77 pg/ml, 0.6 pg/ml and 12.5 pg/ml for TNF-α, TNFR1, TNFR2, and IL-18, respectively.

## Results

### Patient Characteristics

Demographics and clinical characteristics of the 32 patients enrolled in the study are summarized in Table 1. The first cycles commenced between October 2010 and May 2012. Patients were predominantly female (59%), Caucasian (84%) and were on average 55 years old. Of those enrolled, 59% were diagnosed with lymphoma and the remaining 41% had breast cancer. Randomization was considered successful, as there were no significant differences between groups randomized to receive mesna in the first or second cycle regarding gender, race, diagnosis, treatment type and ECOG performance status as well as any of the baseline biomarkers or plasma protein oxidation levels.

**Table 1:** Demographics for randomized subjects N = 32.

	Overall (N = 32)		Mesna: Saline (N = 16)		Saline: Mesna (N = 16)		p-value*
	N	%	N	%	N	%	
**Gender**							0.15
Female	19	59.38	7	43.75	12	75.00	
Male	13	40.63	9	56.25	4	25.00	
**Race**							1.00
White	27	84.30	13	81.25	14	87.50	
Black/African American	5	15.63	3	18.75	2	12.50	
**Ethnicity**							NA
Non-Hispanic	32	100.00	16	100.00	16	100.00	
**Treatment Type**							
AC	11	34.38	5	31.25	6	37.50	1.00
CHOP	19	59.38	10	62.50	9	56.25	
TAC	2	6.25	1	6.25	1	6.25	
**ECOG Performance Status**							0.72
0—Fully Active	18	56.25	8	50.00	10	62.50	
1—Restricted	14	43.75	8	50.00	6	37.50	
**Cancer Diagnosis**							1.00
Breast	13	40.63	6	37.50	7	21.88	
Lymphoma	19	59.38	10	62.50	9	56.25	
**Age** (Mean, Std Dev)	54.78	13.86	51.75	15.51	57.81	11.70	0.22
**BSA** (Mean, Std Dev)	1.97	0.25	1.93	0.26	2.01	0.23	0.35
**Baseline Measures** (Mean, Std Dev)							
Log TNF ALPHA	1.28	1.12	1.30	1.16	1.27	1.11	0.93
Log TNF Receptor 1	7.17	0.46	7.04	0.46	7.29	0.44	0.13
Log TNF Receptor 2	8.23	0.71	8.15	0.74	8.31	0.68	0.51
Log IL-18	6.11	0.84	6.16	0.76	6.06	0.93	0.74
Log PC	4.36	0.36	4.33	0.38	4.39	0.34	0.65
Log Plasma HNE	-0.29	0.21	-0.28	0.20	-0.31	0.22	0.69
Log 3NT	-0.08	0.21	-0.05	0.16	-0.11	0.26	0.41

* p-values calculated using Fisher's exact tests for binary or categorical variables and two-sample t-tests for continuous outcomes

### Biomarker evaluation

The co-administration of mesna with Dox reduced plasma levels of TNF-α, TNFR1 and TNFR2 compared to saline for patients with high baseline values receiving Dox for breast cancer and NHL. There were significant baseline differences between lymphoma versus breast cancer patients for TNF-α, TNFR1, TNFR2 and IL-18. To control for these differences, a linear mixed model found statistically significant differences in these measures over time as well as a baseline-by-treatment interactions. The baseline value was a significant covariate in these models (S2 Table). Furthermore, there was a significant interaction between baseline levels and treatment group indicating that biomarker outcome differences between mesna versus saline groups differed according to baseline levels of TNF-α.

**Fig 3:**
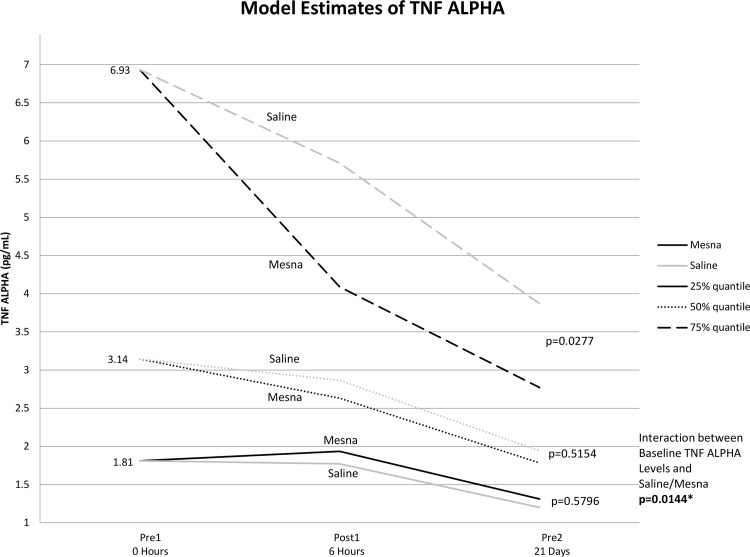
Mean estimates of TNF-α levels for each time point by mesna and saline groups calculated from linear mixed model adjusting for baseline level, time, group (mesna vs saline), chemotherapy regimen and baseline by group interaction.

**Fig 4:**
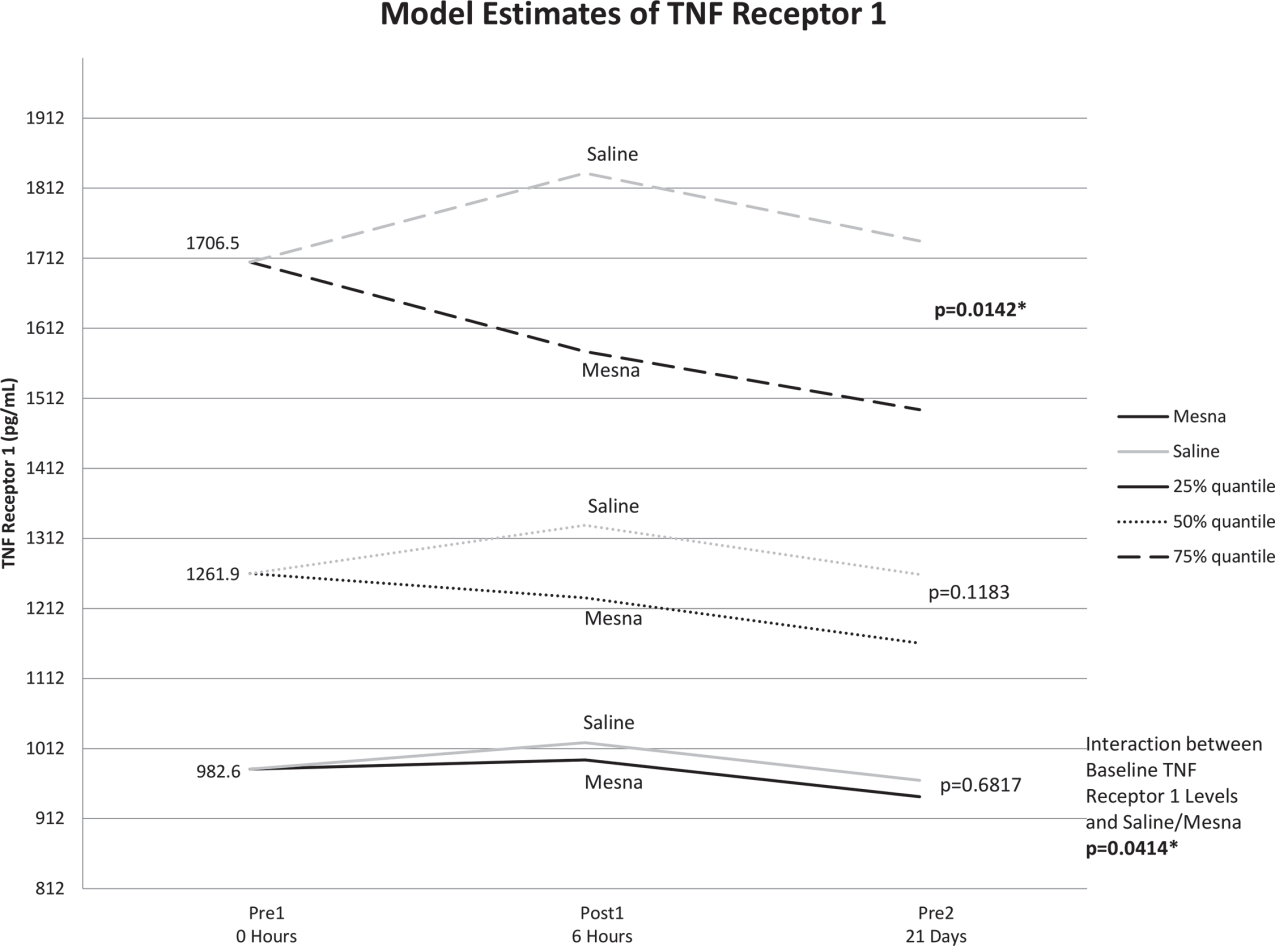
Mean estimates of TNF receptor 1 levels for each time point by mesna and saline groups calculated from linear mixed model adjusting for baseline level, time, group (mesna vs saline), chemotherapy regimen and baseline by group interaction.

**Fig 5:**
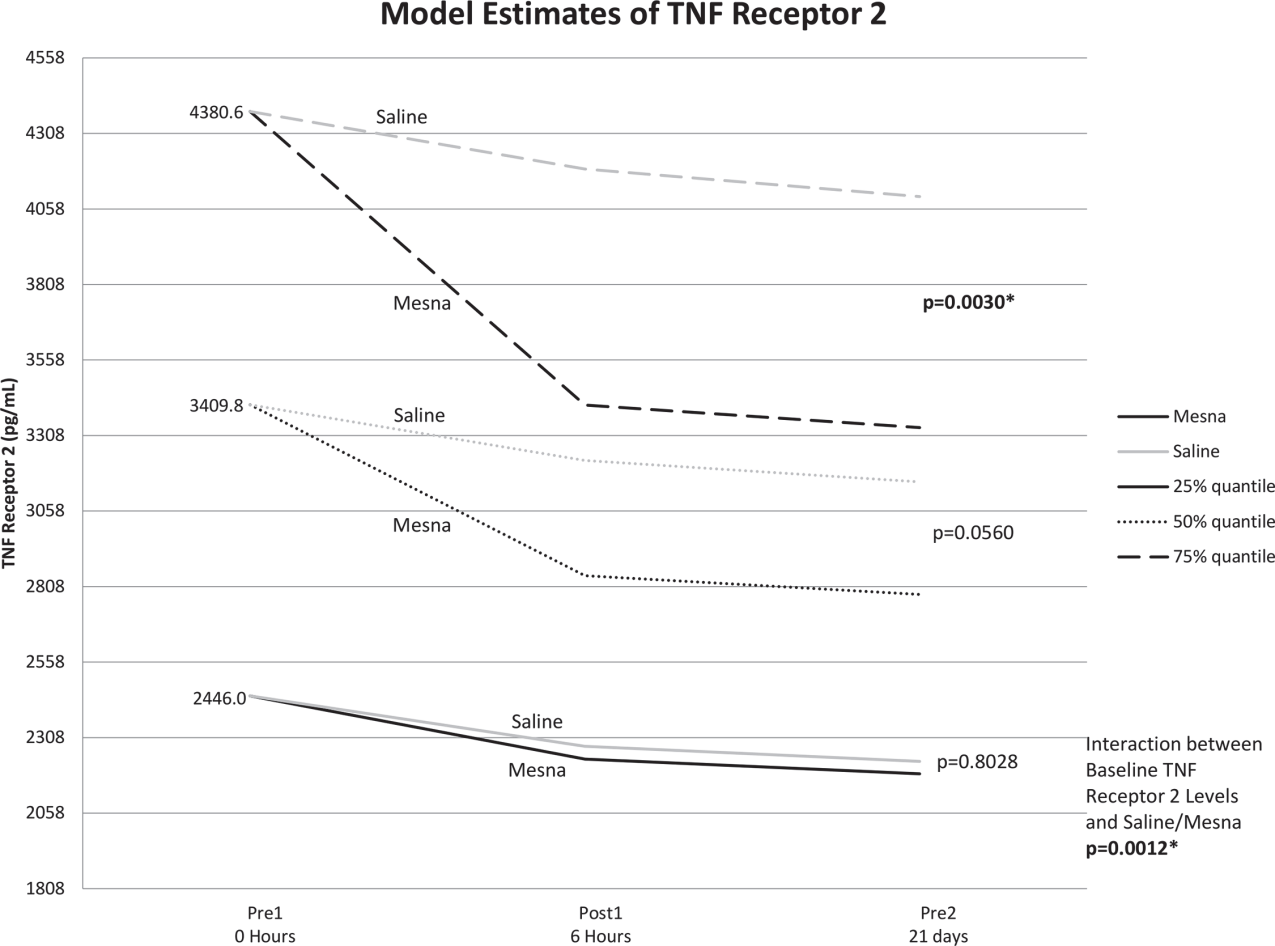
Mean estimates of TNF receptor 2 levels for each time point by mesna and saline groups calculated from linear mixed model adjusting for baseline level, time, group (mesna vs saline), chemotherapy regimen and baseline by group interaction.

To further analyze the baseline by treatment interaction, Bonferonni adjustments for post-hoc comparisons at various baseline levels were considered significant for any p<0.017. As shown in Fig 3, for patients with the 75% percentile level of baseline values or higher, mesna decreased TNF-α levels over time relative to the saline group although not statistically significant (p = 0.028). Similarly, there was a significant reduction in both TNFR1 (Fig 4) and TNFR2 (Fig 5) for the mesna group compared to saline (p = 0.014 and p = 0.003 respectively) in the high baseline cohort. There was also a trend of lower average IL-18 levels for the mesna group compared to saline, although not statistically significant (p = 0.09).

In addition, Table 2 shows that there were significantly positive correlations across TNF-α, TNFR1, TNFR2 and IL-18 (p < 0.0001), suggesting there is a distinct cytokine signature involving activation of the TNF-α signaling pathway. Furthermore, there were also significant correlations over time for the repeated measurements of each of the biochemical measures (S3 Table).

**Table 2:** Spearman Correlations between Baseline Measures.

Correlation (p-value)	Log TNF ALPHA Pre1	Log TNF Receptor 1 Pre1	Log TNF Receptor 2 Pre1	Log IL-18 Pre1	Log PC Naïve Baseline	Log Plasma HNE Naïve Baseline	Log 3NT Naïve Baseline
Log TNF ALPHA Pre1	1.00	**0.71041**	**0.82918**	**0.76613**	0.02163	-0.11107	-0.18585
		**(<.0001)**	**(<.0001)**	**(<.0001)**	(0.9065)	(0.5451)	(0.3085)
Log TNF Receptor 1 Pre1		1.00	**0.89076**	**0.74304**	0.01576	-0.05865	-0.05095
			**(<.0001)**	**(<.0001)**	(0.9318)	(0.7498)	(0.7818)
Log TNF Receptor 2 Pre1			1.00	**0.72654**	-0.04069	-0.13563	-0.10777
				**(<.0001)**	(0.8250)	(0.4592)	(0.5571)
Log IL-18 Pre1				1.00	0.07845	-0.06488	-0.12097
					(0.6696)	(0.7242)	(0.5096)
Log PC Naïve Baseline					1.00	0.24853	-0.33028
						(0.1702)	(0.0649)
Log Plasma HNE Naïve Baseline						1.00	-0.33138
							(0.0639)
Log 3NT Naïve Baseline							1.00

### Plasma protein oxidation or lipid peroxidation

Unlike the findings with cytokine levels, there were no significant baseline differences between lymphoma versus breast cancer patients with PC (77.84 vs. 79.23, p = 0.89), plasma protein-bound HNE (0.71 vs. 0.80, p = 0.13) and 3-NT (0.93 vs. 0.91, p = 0.79). With regards to PC, protein-bound HNE, and 3-NT, no statistically significant differences were observed between the mesna and saline groups at six hours post Dox administration, though a non-statistically significant reduction in PC was observed with mesna (p = 0.20).

### Toxicities

Grade 3 and 4 toxicities are reported in Table 3 and all toxicities regardless of attribution are listed in S4 Table): Toxicities were summarized for each patient by cycle and the treatment received. There were no differences in troponin levels at 6 hours post treatment between the mesna-containing cycles and the saline-containing cycles. Since the mesna vs saline randomization occurred only in the first 2 cycles of therapy, the only cycle that could have ben delayed by mesna was cycle 2 of the patients randomized to mesna in cycle 1, and there were no treatment delays for toxicity among these patients.

**Table 3:** All grade 3 or 4 toxicities split by treatment and cycle.

	Mesna				Saline			
	Cycle 1 (N = 16)		Cycle 2 (N = 16)		Cycle 1 (N = 16)		Cycle 2 (N = 16)	
	N	%	N	%	N	%	N	%
Anemia	0	0%	1	6%	1	6%	1	6%
Blood bilirubin increased	0	0%	0	0%	0	0%	1	6%
Fatigue	0	0%	0	0%	0	0%	1	6%
Febrile neutropenia	0	0%	0	0%	1	6%	1	6%
Headache	2	13%	0	0%	0	0%	0	0%
Hyperglycemia	0	0%	0	0%	1	6%	0	0%
Hypokalemia	0	0%	1	6%	0	0%	0	0%
Hyponatremia	0	0%	0	0%	2	13%	0	0%
Infected Toe	0	0%	0	0%	0	0%	1	6%
Lymphocyte count decreased	2	13%	0	0%	0	0%	3	19%
Nausea	1	6%	0	0%	1	6%	0	0%
Neutrophil count decreased	4	25%	0	0%	5	31%	2	13%
Platelet count decreased	1	6%	0	0%	0	0%	0	0%
Vomiting	0	0%	0	0%	1	6%	0	0%
Weight loss	0	0%	1	6%	0	0%	0	0%
White blood cell decreased	3	19%	2	13%	3	19%	1	6%
Lung infection	0	0%	0	0%	0	0%	1	6%
Total	13		5		15		12	

## Discussion

This study has demonstrated that mesna co-administration decreases TNF-α, TNFR1 and TNFR2 levels after Dox-containing chemotherapy as compared to receiving Dox-containing chemotherapy alone, indicating a potential role for mesna in mediating toxicities resulting from TNF-α signaling during cancer treatment. Furthermore, the finding that this effect is most pronounced in patients with higher baseline levels of TNF pathway-related cytokines suggests that these baseline levels may be biomarkers that can identify patients most likely to benefit from the strategy of rationally administering mesna to patients receiving redox active chemotherapy.

We embarked on the preclinical and clinical evaluation of mesna interaction with Dox in relationship to TNF-α in part because of our initial findings that patients who were receiving chemotherapy who coincidentally received mesna with their chemotherapy had significantly less PC, a marker of oxidative stress (p<0.05) [3]. However, measures of oxidative stress (PC, protein-bound HNE, 3-NT) in this study did not demonstrate a significant reduction with mesna co-administration. One reason for the inability to detect differences in plasma protein oxidation or lipid peroxidation may have been the relatively late time point examined, six hours after Dox administration. It is possible that damaged proteins are rapidly cleared from the circulation and future studies should examine plasma samples obtained closer to the administration of Dox.

In a previous observational clinical study, paired samples from patients receiving Dox found that TNF-α plasma levels are significantly increased at six hours after treatment with Dox [3]. This clinical finding is consistent with our preclinical murine findings that Dox administration causes an increase in plasma TNF-α measured six hours after Dox administration while concomitant treatment with mesna reduces this increase in TNF-α [3]. However, in this current study, mean plasma TNF-α decreased by six-hours post administration of Dox-containing chemotherapy with or without mesna, though participants with higher baseline levels of TNF-α did have a significantly greater reduction at six hours than when receiving concomitant mesna. Interestingly, subjects in this current trial enrolled with elevated pre-treatment plasma TNF-α levels in comparison to levels seen in healthy volunteers [15]. It is possible that pre-treatment TNF-α level is related to tumor burden and that early treatment effect may begin to reduce this TNF-α input shortly upon treatment, which may explain the decline of TNF-α levels in the saline group with the highest baseline values. However, in this study, the inclusion of patients with both lymphoma and breast cancer confounded any determination of the relation of tumor burden to cytokine levels. Future clinical studies may be strengthened by stratification by tumor burden, and preclinical studies using tumor-bearing mice are planned.

Mesna is approved by the U.S. FDA for the prevention of hemorrhagic cystitis after treatment with ifosfamide chemotherapy. Upon intravenous infusion, mesna is rapidly oxidized to mesna disulfide (dimesna). Mesna and dimesna are rapidly filtered and excreted by the kidneys. In the nephron dimesna is reduced back to mesna and in the bladder mesna detoxifies acrolein and other toxic ifosfamide metabolites. Mesna is rapidly filtered by the kidneys and has a very limited volume of distribution [16]. Mesna is not thought to interfere with chemotherapy’s therapeutic efficacy [17] and is routinely coincidentally administered with multi-agent chemotherapy regimens without clinical concern.

In our pre-clinical murine studies, Dox administration resulted in increased brain oxidative stress, even though neither Dox nor its major metabolite cross the blood brain barrier and enter the CNS [4], suggesting that chemotherapy- induced CNS events occur not due to direct toxicity from chemotherapy but via a secondary messenger or downstream event. Similar murine studies conducted with systemic co-administration of TNF-α neutralizing antibodies with chemotherapy found that inhibiting systemic TNF-α prevented the CNS injury associated with systemic chemotherapy administration [5]. Building upon the earlier finding that plasma ApoA1 participates in TNF-α suppression [18], we found that oxidation of plasma ApoA1 impaired its ability to suppress TNF-α secretion by monocytes, and that mesna co-incubation with Dox protected ApoA1 from oxidation and prevented Dox-mediated TNF-α secretion from a macrophage cell line [3].

In the current study, the amount of inflammatory cytokine reduction achieved by co-administration of mesna with chemotherapy positively correlates with the patient’s pre-treatment cytokine levels. As illustrated in Figs 3–5, patients who presented with the lowest quartiles systemic TNF-α TNFR1 and TNFR2 may receive little benefit in terms of cytokine signals with the co-administration of mesna, while those who present with levels at the highest quartile have the largest percent reduction in inflammatory cytokines with mesna co-administration. In recent studies of breast cancer survivors, key polymorphisms in TNF-related signaling pathways correlate with the severity of neurocognitive sequelae [19]. Taken together, these findings reinforce the relevance of the TNF-α pathway and naturally lead to the question of whether cytokine level or related polymorphisms may predict those most at risk to develop CICI and serve as a marker of who may benefit the most from mesna co-administration.

Our study has important limitations. First, though the rationale of the study was based on our preclinical data that link Dox-mediated plasma protein oxidation, TNF-α and cognitive dysfunction, we did not measure any cognitive outcomes in the patients enrolled in the study. Our rationale was that we first wanted to demonstrate that mesna had the postulated biochemical effect on plasma protein oxidation and inflammatory cytokines before exposing a cohort of patients to rationally administered mesna during all cycles of therapy. Also, we did not measure the levels of anti-inflammatory cytokines, which also could play a role in our model of pathophysiology of CICI.

In summary, we have shown that co-administration of mesna during one cycle of Dox-based chemotherapy reduced the post-treatment levels of TNF-α, TNFR1 and TNFR2. Subset analyses further support the additional hypothesis that those patients who present with highest pre-treatment levels of TNF-α, TNFR1 and TNFR2 are the most likely to benefit from mesna co-administration and should be prospectively tested in future studies. Although this study did not directly address the potential clinical role for mesna in the prevention of CICI, it did demonstrate a clinical interaction between mesna and Dox on cytokine levels associated with CICI, and therefore provides a clinical basis for a future mechanistically-based prospective evaluation of mesna for the prevention of CICI.

## Supporting Information

S1 CONSORT Checklist CONSORT Checklist. (PDF)

S1 Dataset (XLSX)

S1 Protocol Study protocol. (PDF)

S1 Table Geometric Means and 95% Exponentiated CI’s for Log Levels for each biochemical marker by Randomization Groups. (DOCX)

S2 Table Parameter estimates from Repeated Measures Models for each measure. (DOCX)

S3 Table Spearman Correlations between timepoints within the same measure. (DOCX)

S4 Table All toxicities regardless of attribution split by treatment and cycle. (DOCX)
