# Supplementary material for: Plasma TNF-α and Soluble TNF Receptor Levels after Doxorubicin with or without Co-Administration of Mesna—A Randomized, Cross-Over Clinical Study
Source: PLoS One. 2015 Apr 24;10(4):e0124988. doi: 10.1371/journal.pone.0124988 (PMC4409356; doi:10.1371/journal.pone.0124988)
Supplement: S1 Protocol — (PDF) [file pone.0124988.s003.pdf]

Local Protocol #: 10-0431-F1V

TITLE: Randomized, blinded trial of Mesna to prevent doxorubicin-induced plasma protein oxidation and TNF-α release

Coordinating Center: Markey Cancer Center
University of Kentucky

***Principal Investigator:** John Hayslip, MD, MSCR
800 Rose St., CC450
Lexington, KY 40536
Tel: 859-257-3379
Fax: 859-257-4450
j.hayslip@uky.edu

Co-Investigators:

Mara Chambers, MD<br>800 Rose St,. CC451<br>Lexington, KY 40536<br>Tel: 859-323-8043<br>Fax: 859-257-4450<br>Mdcham2@email.uky.edu	D. Allan Butterfield, PhD.<br>Dept. of Chemistry, Ctr. of Membrane Sciences, and Sanders-Brown Ctr. on Aging, 500 Rose Street, 121 Chem-Phys Bldg.<br>Tel: 859-257-3184<br>Fax: 859-257-5876<br>dabcns@uky.edu
Jeffrey A. Moscow, MD<br>Department of Pediatrics<br>740 S. Limestone Room J457<br>Lexington, KY 40536<br>Tel: (859) 323-1436<br>Fax: (859) 257-6048<br>jmoscow@ukyedu	Daret St. Clair, PhD.<br>Department of Toxicology<br>458 HSRB<br>Lexington, KY 40536<br>Tel: (859) 257-3956<br>Fax: (859) 323-1059<br>dstcL00@uky.edu
Breast Cancer Care Team:<br>Suleiman Massarweh, MD<br>Edward Romond, MD<br><br>Lymphoma Care Team:<br>Roger Fleischman, MD, PhD.<br>Dianna Howard, MD<br>LazarosLekakis, MD<br>Kevin McDonagh, MD<br>Greg Monohan, MD	Statistician<br>Brent Shelton, PhD., MS<br>2365 Harrodsburg Rd - Suite A230<br>Markey Cancer Control Program<br>Lexington, KY 40504-3381<br>Tel : 859-219-0773(x301)<br>Fax : 859-219-0557<br>bshelton@kcp.uky.edu

Version # / Version Date: #1d 11/15/2011

SCHEMA

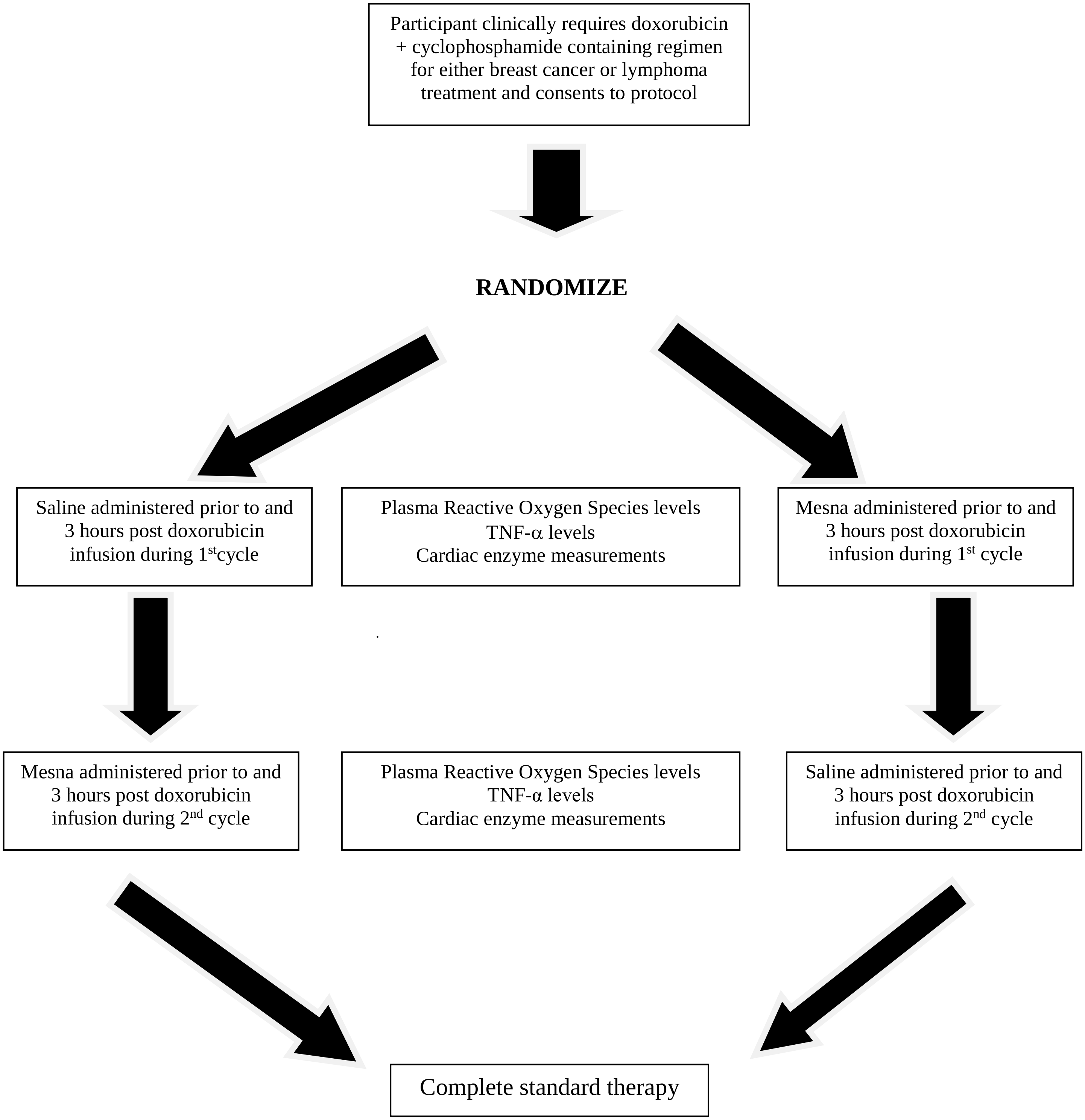
Participant clinically requires doxorubicin + cyclophosphamide containing regimen for either breast cancer or lymphoma treatment and consents to protocol
RANDOMIZE
Saline administered prior to and 3 hours post doxorubicin infusion during 1st cycle
Plasma Reactive Oxygen Species levels
TNF-α levels
Cardiac enzyme measurements
Mesna administered prior to and 3 hours post doxorubicin infusion during 1st cycle
Mesna administered prior to and 3 hours post doxorubicin infusion during 2nd cycle
Plasma Reactive Oxygen Species levels
TNF-α levels
Cardiac enzyme measurements
Saline administered prior to and 3 hours post doxorubicin infusion during 2nd cycle
Complete standard therapy

TABLE OF CONTENTS

Page

SCHEMA i

1. OBJECTIVES 1
- 1.1 **Primary Objectives** 1
- 1.2 **Secondary Objectives** 1

2. BACKGROUND 1
- 2.1 **Chemotherapy Induced Neurocognitive Change 'Chemobrain'** 1
- 2.2 **Mesna** 1
- 2.3 **Standard Chemotherapy Regimens for Breast Cancer and NHL** 2
- 2.4 **Rationale** 3
- 2.5 **Correlative Studies Background** 4

3. PATIENT SELECTION 5
- 3.1 **Eligibility Criteria** 5
- 3.2 **Exclusion Criteria** 6
- 3.3 **Inclusion of Women and Minorities** 6

4. REGISTRATION PROCEDURES 6

5. TREATMENT PLAN 7
- 5.1 **Agent Administration** 7
- 5.2 **General Concomitant Medication and Supportive Care Guidelines** 8
- 5.3 **Duration of Therapy** 9
- 5.4 **Duration of Follow Up** 9
- 5.5 **Criteria for Removal from Study** 9

6. DOSING DELAYS/DOSE MODIFICATIONS 9

7. ADVERSE EVENTS: LIST AND REPORTING REQUIREMENTS 10
- 7.1 **Comprehensive Adverse Events and Potential Risks Lists (CAEPRs)** 10
- 7.2 **Adverse Event Characteristics** 11
- 7.3 **Expedited Adverse Event Reporting** 12
- 7.4 **Routine Adverse Event Reporting** 13

8. PHARMACEUTICAL INFORMATION 13

9. RESEARCH STUDIES 14
- 9.1 **Laboratory Studies** 14

10. STUDY CALENDAR 15

11. DATA REPORTING / REGULATORY CONSIDERATIONS 16
- 11.1 **Data Reporting** 16

12. STATISTICAL CONSIDERATIONS 16

REFERENCES 18

APPENDICES

APPENDIX A
Declaration of Helsinki 20

APPENDIX B
Mesna Package Insert 24

1. OBJECTIVES

1.1. Primary Objectives

- Determine if mesna treatment significantly reduces plasma protein oxidation and TNF-α levels in patients receiving doxorubicin containing chemotherapy

1.2. Secondary Objectives

- Determine if mesna co-administration with anthracycline prevents cardiac myocyte injury

2. BACKGROUND

2.1 **‘Chemobrain’**

‘Chemobrain’ and ‘chemo-fog’ are loose, but commonly used, terms that refer to cognitive dysfunction after chemotherapy. While the mechanisms of cognitive dysfunction after central nervous system (CNS)-directed therapy are widely accepted and understood, the risks of cognitive impairment after cancer therapies that do not directly involve the CNS are less well accepted or understood. Some of the most perplexing situations are the experiences of patients who report cognitive impairment after receiving the drug doxorubicin, which does not enter the CNS.

Postulated mechanisms for inducing ‘chemobrain’ have been recently reviewed but are not well agreed upon (1). We have proposed a model of doxorubicin-induced CNS and cardiac damage, in which a TNF-α-mediated process is initiated by the direct intravascular oxidative modification of plasma proteins by doxorubicin, followed by release of TNF-α, with tissue damage then resulting as a downstream effect of TNF-α. These high levels of TNF-α cross the blood-brain barrier and may initiate a cascade of events that results in the clinical syndrome referred to as ‘chemobrain’. We hypothesize that preventing doxorubicin induced plasma protein oxidation may prevent the initiation of this cascade thereby preventing ‘chemobrain’ without interfering with the efficacy of the chemotherapy.

2.2 **Mesna**

Mesna is approved by the FDA for the prevention of hemorrhagic cystitis after treatment with ifosfamide (Ifosfamide Package Insert). In humans, mesna, sodium-2-mercaptoethane sulfonate, is rapidly oxidized to mesna disulfide (dimesna). Dimesna is rapidly filtered and excreted by the kidneys. In the nephron, dimesna is reduced to mesna and mesna detoxifies acreolin and other toxic ifosfamide metabolites. Despite numerous xenograft studies to assess the potential that this reduction in bladder toxicity may be associated with a reduction in treatment efficacy, no impairment of the treatment efficacy has been seen even when mesna was tested at 20-fold higher doses.

The serum half-life of mesna is 4-8 hours (mesna package insert). Mesna remains almost

entirely intravascular until filtered by the kidneys. A typical dosing schedule of mesna with ifosfamide is 240 mg/m^2 at 0, 4, and 8 hours after receiving ifosfamide. This is rational because the half-life of ifosfamide is 5-6 hours and mesna must inhibit ifosfamide metabolite injury in the bladder. In the current study, our model suggests mesna will inhibit doxorubicin induced, TNF-α mediated injury. Although doxorubicin has multi-phasic pharmacokinetics, the serum clearance half-life is approximately five minutes. Accordingly, we hypothesize that two doses spaced three hours apart will be both safe and effective at reducing intravascular reactive oxygen species. Therefore this study will use the standard dose of 720 mg/m^2, typically divided into three equal doses with ifosfamide, but here divided into two equal doses with doxorubicin.

2.3 Standard Chemotherapy Regimens for Breast Cancer and NHL

The primary hypothesis of this study is that mesna will inhibit doxorubicin-induced reactive oxygen species (ROS) preventing TNF-α production and reducing the neuro-cognitive effects of doxorubicin-containing chemotherapy. Doxorubicin is almost never used clinically as a single-agent and is almost always given in combination with other drugs. In this study, we will focus on doxorubicin-containing regimens used for the treatment of breast cancer and non-Hodgkin lymphoma.

The treatment of breast cancer is complex and treatment recommendations are individualized based upon patient and tumor characteristics. Many standard breast cancer chemotherapy treatments include a doxorubicin-cyclophosphamide combination as a key element of the treatment. In some instances, although increasingly uncommon, doxorubicin and cyclophosphamide is all that is required. More commonly, treatments with doxorubicin and cyclophosphamide are administered for approximately four treatments and then followed with additional treatment with paclitaxel or docetaxel. In some instances, the monoclonal antibody trastuzomab is also required as part of standard-of-care treatment for breast cancer. There are other instances in which clinical judgment dictates that other combinations should be used in place of doxorubicin-cyclophosphamide. Given the complex and individualized decision making in breast cancer care, this protocol does not seek to define which patients are best offered treatment with a doxorubicin-cyclophosphamide containing regimen. Instead, this study will enroll patients whose oncologist has suggested that the standard doxorubicin-cyclophosphamide chemotherapy cassette is at least a component of the overall recommended treatment. This recommendation is made regardless a patient's interest in participating in this trial evaluating the use of mesna.

Similarly, the treatment of non-Hodgkin lymphoma (NHL) is complex and based upon the subtype of NHL, patient characteristics, and if the patient has received previous treatments for NHL. The most commonly prescribed treatment regimen for NHL is a combination of cyclophosphamide, doxorubicin, vincristine, and prednisone with or without rituximab referred to as CHOP +/- R. This study does not seek to define the standard-of-care for all types and presentations of NHL. Instead, participants are only eligible to be considered for this study after the determination has been made that CHOP +/- rituximab is their treatment recommendation regardless the participant's interest in the current study evaluating the addition of mesna.

2.4 Rationale

The basis of this proposal is our recent discovery of increased oxidative stress (an excess of free radical production over free radical scavenging) in the brains of mice treated intraperitoneally (i.p.) with doxorubicin(2). Increased markers of protein oxidation and lipid peroxidation were found in the brain even though doxorubicin and its major metabolite, doxorubicinol (Dox-ol), do not cross the blood brain barrier (BBB). Rather, doxorubicin causes oxidative modification of plasma proteins, leading to elevation of circulating TNF-α, which enters the brain to reside in neurons (and likely other cells) to induce mitochondrial damage, loss of function, and apoptosis. Anti-TNF-α antibody blocked these effects in the brain, consistent with the notion that blocking TNF-α entry to the brain prevents brain mitochondrial dysfunction and apoptosis(3-5). Consistent with a role for the mitochondria in brain-resident oxidative stress following doxorubicin treatment, doxorubicin leads to oxidative dysfunction of mitochondrial-resident MnSOD. Moreover, doxorubicin leads to loss of brain glutathione (GSH), consistent with an oxidative environment in the brain, whereas elevation of GSH protected against doxorubicin-induced oxidative stress in the brain(2).

To determine whether the oxidative events detected in animal plasma and tissues can be found in patients during doxorubicin administration, we conducted a clinical study that examined protein oxidative modification in 12 children receiving multi-agent chemotherapy regimens that contained doxorubicin. We found that children receiving coincidental mesna (n=5) showed a decrease in plasma protein-bound HNE (a product of free radical-induced lipid peroxidation that covalently binds to proteins), whereas children not receiving coincidental mesna (n=7) showed an increase in plasma protein HNE. Therefore, this initial clinical study underscores the clinical merit of our underlying hypothesis: doxorubicin administration causes oxidation of plasma proteins, which is the first step in TNF-α-mediated damage to normal tissues, and that mesna may offer protection against this process.

Clinically, mesna is administered with ifosfamide and cyclophosphamide to prevent hemorrhagic cystitis caused by acrolein, a product of lipid peroxidation. Mesna is thought to have little cellular uptake, with a purported mechanism of action that is extracellular in nature. Mesna is widely used in chemotherapy regimens, and it is commonly accepted that mesna does not impact the effectiveness of chemotherapy. Mesna has previously been assumed to have no interaction with doxorubicin. However, since mesna is an anti-oxidant and chemically similar to γ-GCEE, which prevents doxorubicin-induced brain damage (2), it seemed possible mesna could prevent doxorubicin-induced tissue damage in a model of tissue damage where the initiating step was the extracellular and intravascular oxidative damage of plasma proteins.

Further preclinical studies in mice have demonstrated, in fact, that mesna protects against doxorubicin-induced plasma protein oxidation and abrogates expression of markers of CNS and cardiac tissue injury after doxorubicin administration (Butterfield, St. Clair, unpublished data). Furthermore, the Butterfield lab has demonstrated a direct interaction between Mesna and doxorubicin. Using the dichlorofluorescein (DCF) assay to measure the free radical scavenging ability of mesna in the presence of doxorubicin, Figure 1 shows that doxorubicin caused a significant increase in DCF fluorescence, indicating an

increase in free radical production. When mesnawas co-administered with doxorubicin, DCF fluorescence was significantly suppressed relative to doxorubicin alone. The capability of mesna to scavenge free radicals, particularly in the plasma, may be similar mechanistically to glutathione (GSH), as mesna is oxidized to dimesna in the blood. The donation of H· from the sulfhydryl group of mesna upon oxidation may quench free radicals produced by doxorubicin, thus affording biomolecules protection from ROS damage. These studies add further support to the hypothesis that mesna can prevent doxorubicin-generated injury to normal tissues.

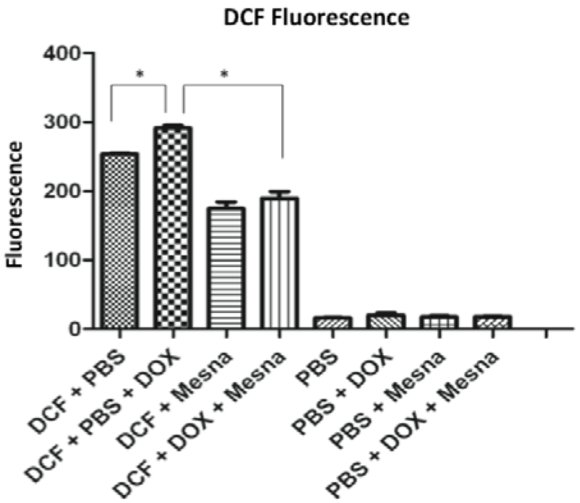

Figure 1. Reactive oxygen species generation by doxorubicin and radical scavenging ability of mesna.Dichlorofluoroscein (DCF) assay was employed to measure production of free radicals, which oxidize dichlorofluorescin (DCFH) to dichlorofluorescein (DCF). DCF is highly fluorescent and can be detected using a spectrofluorometer (λex= 495 nm, λem= 530 nm). Left panel: DOX (250 nM) causes a significant increase in DCF fluorescence, and co administration of mesna (25 µg/mL) with doxorubicin significantly suppresses this effect relative to doxorubicin alone (*p<0.0001, n=3 per group). DOX and mesna alone or together do not contribute to signal intensity.

2.5 Correlative Studies Background

Subjects will be assessed for both biochemical/molecular evidence of doxorubicin-induced cardiovascular disease with biomarkers of cardiomyotoxicity. To date, the most reliable cardiac markers used for the detection of cardiomyocyte necrosis are cardiac troponin T (cTnT) and cardiac troponin I (cTnI) (21-23). Of note, the timing of blood sampling for cardiac markers has not been routinely established. Atrialnatriuretic peptides (ANP), brain natriuretic peptides (BNP), and N-terminal-pro-brain natriuretic peptides (NT-proBNP) have all been measured with variant outcomes. The most reliable indicators of myocardial toxicity to date, in association with noninvasive echo parameters, include BNP and NT-proBNP(23, 24). Therefore, cTnI, and BNP levels will be determined during cycles 1 and 2 at Hr 0 and at 6 hour post starting doxorubicin infusion.

3. PATIENT SELECTION

3.1 Eligibility Criteria

3.1.1 Participants must have histologically or cytologically confirmed breast cancer or non-hodgkin lymphoma and independent of protocol eligibility be determined to require one of the chemotherapy regimens listed in 3.1.2.

3.1.2 Participants must require as standard-of-care treatment a chemotherapy regimen that includes one of the following combinations:

- doxorubicin 60 mg/m^2 and cyclophosphamide 600 mg/m^2;
- doxorubicin 50 mg/m^2, cyclophosphamide 500 mg/m^2, and docetaxel 75 mg/m^2;
- doxorubicin 50 mg/m^2, cyclophosphamide 750 mg/m^2, vincristine 1.4 mg/m^2 (capped at 2 mg dose), and prednisone 100 mg +/- rituximab 375 mg/m^2

3.1.3 Age ≥18 years.

Because these treatment regimens are rarely used in pediatric oncology, children are excluded from this study but will be eligible for future pediatric phase 2 trials.

3.1.4 Life expectancy of greater than 6 months.

3.1.5 Zubrod performance status 2 or better.

3.1.6 Patients must have normal organ and marrow function as defined below:

-	leukocytes	≥3,000/mcL (unless due to cancer in marrow)
-	absoluteneutrophil count	≥1,500/mcL (unless due to cancer in marrow)
-	platelets	≥100,000/mcL (unless due to cancer in marrow)
-	total bilirubin	≤1.5 X normal institutional limits
-	AST(SGOT)/ALT(SGPT)	≤2.5 X institutional upper limit of normal
-	creatinine	within normal institutional limits
	OR	
-	creatinine clearance	≥60 mL/min/1.73 m^2 for patients with creatinine levels above institutional normal
-	left ventricular function	≥ 50 % ejection fraction

3.1.7 Because the standard of care chemotherapy agents are known to be teratogenic, women of child-bearing potential and men must agree to use adequate contraception (hormonal or barrier method of birth control; abstinence) prior to study entry and for the duration of study participation. Should a woman become pregnant or suspect she is pregnant while

participating in this study, she should inform her treating physician immediately.

3.1.8 Ability to understand and the willingness to sign a written informed consent document.

3.2 Exclusion Criteria

3.2.1 Patients who have had chemotherapy or radiotherapy within 4 weeks (6 weeks for nitrosoureas or mitomycin C) prior to entering the study or those who have not recovered from adverse events due to agents administered more than 4 weeks earlier.

3.2.2 Patients may not be receiving any other investigational agents.

3.2.3 Patients with known brain metastases should be excluded from this clinical trial because progressive neurologic dysfunction would confound the evaluation of neuro-cognitive outcomes.

3.2.4 Patients requiring ongoing pharmacologic treatment of dementia are excluded.

3.2.5 Uncontrolled intercurrent illness including, but not limited to, ongoing or active infection, symptomatic congestive heart failure, unstable angina pectoris, cardiac arrhythmia, or psychiatric illness/social situations that would limit compliance with study requirements.

3.2.6 Pregnant women are excluded from this study because the chemotherapy agents have known teratogenic or abortifacient effects. Because there is a potential risk for adverse events in nursing infants secondary to treatment of the mother with chemotherapy, breastfeeding should be discontinued if the mother is treated with chemotherapy.

3.2.7 HIV-positivity is NOT a specific exclusion criteria.

3.3 Inclusion of Women and Minorities

Both men and women and members of all races and ethnic groups are eligible for this trial.

4. REGISTRATION PROCEDURES

Informed consent must be documented by the PI or an IRB approved designate by signing an IRB approved consent form prior to initiating any study procedures.

5. TREATMENT PLAN

5.1 Agent Administration

Treatment will typically be administered on an outpatient basis. Reported adverse events and potential risks for mesna are described in Section 7. Appropriate dose modifications for mesna and other agents are described in Section 6. No investigational or commercial agents or therapies other than those described below shouldbe administered with the intent to treat the patient's malignancy. Pre-printed chemotherapy order sets are provided for each of the approved regimens including required pre-medications.

**"AC"**					
**Agent**	**Premedications**	**Dose**	**Route**	**Schedule**	**Cycle Length**
Mesna or saline control	N/A	360 mg/m^2 in 50 mL NS	IV over 15minutes. Give after first correlative lab sample has been obtained and **before** doxorubicin. Give again 3 hours after doxorubicin is started.	Day 1	14-21 days
Doxorubicin	Ondansetron 8 mg orally, dexamethasone 12 mg orally, Aprepitant 125 mg orally	60 mg/m^2	IV over 15 minutes. Give after mesna or saline. Premeds may be given before or during mesna infusion.	Day1	
Cyclophosphamide		600 mg/m^2	IV over 30 minutes. Start 6 hours **after** doxorubicin started and after the second correlative blood sample has been obtained.	Day 1	
May be followed by a taxane and/or trastuzomab-containing regimen and with/without colony stimulating factors in accordance with national or institutional guidelines					

TAC					
**Agent**	**Premedications; Precautions**	**Dose**	**Route**	**Schedule**	**Cycle Length**
Mesna or saline control	N/A	360 mg/m^2 in 50 mL NS	IV over 15minutes. Give after first correlative lab sample has been obtained and **before** doxorubicin. Give again 3 hours after doxorubicin is started.	Day 1	21 days
Doxorubicin	Ondansetron 8 mg orally, dexamethasone 12 mg orally, Aprepitant 125 mg orally	50 mg/m^2	IV over 15 minutes. Give after mesna or saline. Premeds may be given before or during mesna infusion.	Day1	
Cyclophosphamide		500 mg/m^2	IV over 30 minutes. Start 6 hours **after** doxorubicin started and after the second correlative blood sample has been obtained.	Day 1	
Docetaxel		75 mg/m^2	IV over 1 hour. May start immediately after cyclophosphamide.	Day 1	
Pegfigastrim		6 mg	SQ	Day 2	

CHOP +/- Rituximab					
Agent	***Premedications; Precautions***	***Dose***	***Route***	***Schedule***	***Cycle Length***
Mesna or saline control	N/A	360 mg/m^2 in 50 mL NS	IV over 15minutes. Give after first correlative lab sample has been obtained and **before** doxorubicin. Give again 3 hours after doxorubicin is started.	Day 1	14-21 days
Doxorubicin	Ondansetron 16 mg orally	50 mg/m^2	IV over 15 minutes. Give after mesna or saline. Premeds may be given before or during mesna infusion.	Day1	

Cyclophosphamide		750 mg/m^2	IV over 30 minutes. Start 6 hours **after** doxorubicin started and after the second correlative blood sample has been obtained.	Day 1	
Vincristine		1.4 mg/m^2 (capped at 2 mg maximal dose)	IV over 10 minutes after cyclophosphamide	Day 1	
Prednisone		100 mg	Orally, may take first dose in chemo room with ondansetron or at home.	Days 1to 5	
Rituximab		375 mg/m^2	IV	Day 2	
May be administered with growth factor at the treating physician's discretion in accordance with national or institutional guidelines					

5.2 General Concomitant Medication and Supportive Care Guidelines

The case report form must capture the concurrent use of all other drugs, over-the-counter medications, or alternative therapies.

5.3 Duration of Therapy

In the absence of treatment delays due to adverse events, treatment may continue until one of the following criteria applies:

- X Disease progression,
- X Intercurrent illness that prevents further administration of treatment,
- X Unacceptable adverse events(s),
- X Patient decides to withdraw from the study, or
- X General or specific changes in the patient's condition render the patient unacceptable for further treatment in the judgment of the investigator
- X Participant completes the planned treatment course. After completing the first two randomized cycles of therapy, participants will continue to complete their chemotherapy according to standard of care.

5.4 Duration of Follow Up

Patients will be followed until they initiate their third cycle of chemotherapy or until death, whichever occurs first for the collection of study data and will be followed as per routine standard of care thereafter. Patients removed from study for unacceptable adverse events will be followed until resolution or stabilization of the adverse event.

5.5 Criteria for removal from Study

Patients will be removed from study when any of the criteria listed in Section 5.3 applies. The reason for study removal and the date the patient was removed must be documented in the Case Report Form.

6. DOSING DELAYS/DOSE MODIFICATIONS

Clinically significant adverse reactions to mesna are quite uncommon and the symptoms may be indistinguishable from the symptoms of the concomitantly administered chemotherapy. One rare but potentially clinically significant mesna toxicity is hypersensitivity in those individuals allergic to sulfur compounds. Accordingly, any participant who exhibits grade III hypotension, facial edema, anaphylaxis, or other severe allergic symptoms in the investigators opinion, that aren’t attributed to another treatment in the regimen will be removed from study and will not receive future mesna.

Prior to beginning each cycle of therapy the following criteria must be reviewed and treatment should be delayed unless in the opinion of the treating physician and participant it is in the participant's best clinical interest to continue with treatment on the pre-planned schedule:

- ANC ≥ 1,500 (unless due to tumor infiltration in marrow)
- Platelets ≥ 100,000 (unless due to tumor infiltration in marrow)
- Any non-hematologic toxicities should be recovered to grade 1 or better

Participants may be delayed from initiating a subsequent cycle for up to 3 weeks. In the event a participant has not recovered after 3 weeks, participants should be removed from study and the PI contacted within 24 hours.

If drugs other than mesna (ie chemotherapy) are thought to be causing unacceptable toxicities dose reductions will be made according standard clinical practice. The rationale for proceeding with treatment despite low ANC, low platelets, persistent toxicities, or dose adjustments should be recorded in the participant's clinical record.

7. ADVERSE EVENTS: LIST AND REPORTING REQUIREMENTS

Adverse event (AE) monitoring and reporting is a routine part of every clinical trial. The following list of AEs (Section 7.1) and the characteristics of an observed AE (Section 7.2) will determine whether the event requires expedited (via Oncore) reporting **in addition** to routine reporting (section 7.3).

7.1 Comprehensive Adverse Events and Potential Risks Lists (CAEPRs)

7.1.1 CAEPRs for Mesna

The Comprehensive Adverse Event and Potential Risks list (CAEPR) provides a single, complete list of reported and/or potential adverse events (AE) associated with an agent. In addition to the comprehensive list, a subset, the Agent Specific Adverse Event List (ASAEL), appears in a separate column and is identified with ***bold*** and ***italicized*** text. This subset of AEs (the ASAEL) contains events that are considered 'expected' for expedited reporting purposes only.

7.1.1.1 CAEPR for mesna

Because mesna is typically given as a supportive treatment for patients receiving ifosfamide, previous toxicity assessments include the inherent toxicity of ifosfamide.

Table 3

Incidence of Adverse Events and Incidence of Most Frequently Reported Adverse Events in Controlled Studies

Mesna Regimen	IV-IV-IV	IV-Oral-Oral
N exposed<br>Incidence of AEs	119 (100.0%)<br>101 (84.9%)	119 (100.0%)<br>106 (89.1%)
Most Frequently Reported Adverse Events (Preferred Terms)		
	N (%)	N (%)
Nausea	65 (54.6)	64 (53.8)
Vomiting	35 (29.4)	45 (37.8)
Constipation	28 (23.5)	21 (17.6)
Leukopenia	25 (21.0)	21 (17.6)
Fatigue	24 (20.2)	24 (20.2)
Fever	24 (20.2)	18 (15.1)
Anorexia	21 (17.6)	19 (16.0)
Thrombocytopenia	21 (17.6)	16 (13.4)
Anemia	20 (16.8)	21 (17.6)
Granulocytopenia	16 (13.4)	15 (12.6)
Asthenia	15 (12.6)	21 (17.6)
Abdominal Pain	14 (11.8)	18 (15.1)
Alopecia	12 (10.1)	13 (10.9)
Dyspnea	11 (9.2)	11 (9.2)
Chest Pain	10 (8.4)	9 (7.6)
Hypokalemia	10 (8.4)	11 (9.2)
Diarrhea	9 (7.6)	17 (14.3)
Dizziness	9 (7.6)	5 (4.2)
Headache	9 (7.6)	13 (10.9)
Pain	9 (7.6)	10 (8.4)
Sweating Increased	9 (7.6)	2 (1.7)
Back Pain	8 (6.7)	6 (5.0)
Hematuria*	8 (6.7)	7 (5.9)
Injection Site Reaction	8 (6.7)	10 (8.4)
Edema	8 (6.7)	9 (7.6)
Edema Peripheral	8 (6.7)	8 (6.7)
Somnolence	8 (6.7)	12 (10.1)
Anxiety	7 (5.9)	4 (3.4)
Confusion	7 (5.9)	6 (5.0)
Face Edema	6 (5.0)	5 (4.2)
Insomnia	6 (5.0)	11 (9.2)
Coughing	5 (4.2)	10 (8.4)
Dyspepsia	4 (3.4)	6 (5.0)
Hypotension	4 (3.4)	6 (5.0)
Pallor	4 (3.4)	6 (5.0)
Dehydration	3 (2.5)	7 (5.9)
Pneumonia	2 (1.7)	8 (6.7)
Tachycardia	1 (0.8)	7 (5.9)
Flushing	1 (0.8)	6 (5.0)

* All grades

Mesna package insert from controlled studies of Mesna + Ifosfamide

In addition, in post marketing reports, patients who received chemotherapy regimens that contained mesna have reported the following findings that in the opinion of their treating physician may have reasonably been related to the mesna: allergic reactions, hypertension, hypotension, tachycardia, increased liver enzymes, injection site reactions, limb pain, malaise, myaligia, EKG ST-segment elevation, and tachypnea (mesna package insert). In general, most of these adverse reactions were thought to represent an allergic or hypersensitivity reaction to mesna.

7.2 Adverse Event Characteristics

- **CTCAE term (AE description) and grade:** The descriptions and grading scales found in the revised NCI Common Terminology Criteria for Adverse Events (CTCAE) version 4.0 will be utilized for AE reporting. A copy of the CTCAE version 4.0 can be downloaded from the CTEP web site (http://ctep.cancer.gov) for free.

- **‘Expectedness’**: AEs can be ‘Unexpected’ or ‘Expected’ (see Section 7.1 above) for expedited reporting purposes only. ‘Expected’ AEs (the ASAEL) are ***bold and italicized*** in the CAEPR (Section 7.1.1).

- **Attribution** of the AE:
  - Definite – The AE *is clearly related* to the study treatment.
  - Probable – The AE *is likely related* to the study treatment.
  - Possible – The AE *may be related* to the study treatment.
  - Unlikely – The AE *is doubtfully related* to the study treatment.
  - Unrelated – The AE *is clearly NOT related* to the study treatment.

7.3 Expedited Adverse Event Reporting

7.3.1 Expedited AE reporting for this study will use the *Oncore* data management system.

7.3.2 **Expedited Reporting Guidelines** –Reporting Requirements for Adverse Events that occur within 30 Days[1] of the Last Dose of mesna

	**Grade 1**	**Grade 2**	**Grade 2**	**Grade 3**		**Grade 3**		**Grades 4 & 5[2]**	**Grades 4 & 5[2]**
	**Unexpected and Expected**	**Unex-pected**	**Expected**	**Unexpected**		**Expected**		**Unex-pected**	**Expected**
				**with Hospitali-zation**	**withoutHospitali-zation**	**with Hospitali-zation**	**withoutHospitali-zation**		
**Unrelated Unlikely**	Not Required	Not Required	Not Required	10 Calendar Days	Not Required	10 Calendar Days	Not Required	10 Calendar Days	10 Calendar Days
**Possible Probable Definite**	Not Required	10 Calendar Days	Not Required	10 Calendar Days	10 Calendar Days	10 Calendar Days	Not Required	24-Hour; 5 Calendar Days	10 Calendar Days

[1] **Adverse events with attribution of possible, probable, or definite that occur <u>greater</u> than 30 days after the last dose of treatment with mesna require reporting as follows:**

24-hour notification followed by complete report within 5 calendar days for:
- Grade 4 and Grade 5 unexpected events

10 calendar day report:
- Grade 3 unexpected events with hospitalization or prolongation of hospitalization
- Grade 5 expected events

[2] Although an24-hour notification is not required for death clearly related to progressive disease, a full report is required as outlined in the table.

Note: All deaths on study require both routine and expedited reporting regardless of causality. Attribution to treatment or other cause must be provided.

- Expedited AE reporting timelines defined:
  - “24 hours; 5 calendar days” – The investigator must initially report the AE via Oncore within 24 hours of learning of the event followed by a complete Oncore report within 5 calendar days of the initial 24-hour report.
  - “10 calendar days” - A complete Oncore report on the AE must be submitted within 10 calendar days of the investigator learning of the event.

- Any medical event equivalent to CTCAE grade 3, 4, or 5 that precipitates hospitalization (or prolongation of existing hospitalization) must be reported regardless of attribution and designation as expected or unexpected with the exception of any events identified as protocol-specific expedited adverse event reporting exclusions.

- Any event that results in persistent or significant disabilities/incapacities, congenital anomalies, or birth defects must be reported via Oncore if the event occurs following treatment with mesna

7.3.3 Protocol-Specific Expedited Adverse Event Reporting Exclusions

- For this protocol only, certain AEs/grades are exceptions to the Expedited Reporting Guidelines and do not require expedited reporting. The following AEs must be reported through the routine reporting mechanism (Section 7.4):

CTCAE Category	Adverse Event	Grade
Blood and Lymphatic disorders	Anemia	3
Blood and Lymphatic disorders	Febrile neutropenia	3
Immune system disorders	Anaphylaxis	3
Immune system disorders	Cytokine release syndrome	3
Investigations	Lymphocyte count decreased	4
Investigations	Neutrophil count decreased	4
Investigations	Platelet count decreased	3

7.4 Routine Adverse Event Reporting

All Adverse Events **must** be reported in routine study data submissions.

8. PHARMACEUTICAL INFORMATION

A list of the adverse events and potential risks associated with mesna can be found in Section 7.1.

All medications are clinically available and the mesna package insert is attached to this protocol.

9. RESEARCH STUDIES

9.1 Laboratory Studies

9.1.1 <u>Oxidized serum proteins and TNF-α studies</u>

9.1.1.1 Collection of Specimens

A blood sample will be obtained immediately prior to mesna and 6 hours after the first two doses of doxorubicin begin. The blood will be drawn into three 8 mL EDTA-containing tubes at each time point. Paired (pre- and post-doxorubicin) samples for oxidative stress analyses will be obtained twice for each subject, prior to and during the first and second cycles.

9.1.1.2 Handling of Specimens

The blood will be placed on ice as soon as possible after being drawn, and the plasma will be prepared within 30 min. of being drawn to ensure as little modification of plasma proteins as possible (28). Once the plasma samples have been prepared, they will be snap frozen and stored in -80°C freezers for later analyses of oxidative stress parameters.

9.1.1.3 Site Performing Correlative Study

Dr. Butterfield's lab will use well-established methods, which have been routinely performed by the lab personnel, for sample collection and preparation that minimize *in vitro* oxidation of proteins(2, 29).
Dr. St. Clair's lab will measure serum TNF-α levels by enzyme-linked immunosorbent assay as per manufacturers' instructions (TNF-α/TNFSF1A immunoassay, R&D Systems, Minneapolis, MN). Dr. St. Clair's lab will also freeze and store a small quantity of plasma. The stored plasma sample will be identified with study participant number and time-point within protocol drawn, but no personal health information will be included on those tubes. It is hoped that subsequent funding may allow further evaluation of markers of neuronal injury on these plasma samples. No DNA containing elements will be stored.

9.1.2 <u>Doxorubicin-associated cardiac stress</u>

9.1.2.1 Collection of Specimens

cTnI and BNP levels will be determined during cycles 1 and 2 before mesna infusion and at 6 hour post initiating doxorubicin infusion.

9.1.2.2 Handling of Specimens(s)

Blood samples will be collected in approved vacutainer tubes, labeled with time, date, patient name and medical record.

9.1.2.3 Shipping of Specimen(s)

Samples are sent at room temperature to the UK's clinical laboratory.

9.1.2.4 Site(s) Performing Correlative Study
UK pathology.

10. STUDY CALENDAR

Clinical laboratory evaluations are to be conducted within 1 week prior to start of treatment.

	Pre-Study	Cycle 1	Cycle 2	Cycles 3+
Chemotherapy		X	X	Per standard of care
Informed consent	X			Per standard of care
Demographics	X			Per standard of care
Medical history	X			Per standard of care
Concurrent meds	X		X	Per standard of care
Physical exam	X	X	X	Per standard of care
Vital signs	X		X	Per standard of care
Height	X		X	Per standard of care
Weight	X		X	Per standard of care
Performance status	X			Per standard of care
CBC w/diff, plts	X	X	X	Per standard of care
Serum chemistry	X	X	X	Per standard of care
Adverse event evaluation		X	X	Per standard of care
B-HCG	X			
Serum correlative tests		X	X	

11. DATA REPORTING / REGULATORY REQUIREMENTS

Adverse event lists, guidelines, and instructions for AE reporting can be found in Section 7.0 (Adverse Events: List and Reporting Requirements).

11.1 Data Reporting

11.1.1 Method

All data will be reported using the University of Kentucky's Markey Cancer Center's Oncore data system.

11.1.2 Data and Safety Monitoring

The Lucille P. Markey Cancer Center Quality Assurance Committee (QAC) will serve as the data safety monitoring board for this study. The PI is responsible for ensuring that study data is supplied to the QAC as requested. The PI, in accordance with cancer center policies, is responsible for data management and quality.

12. STATISTICAL CONSIDERATIONS

12.1 Study Design/Analysis of Primary Endpoints

This is a stratified, blinded, randomized, cross-over trial of mesna vs. saline control concomitantly with doxorubicin-based chemotherapy. The primary endpoint is to determine whether mesna prevents a doxorubicin-induced increase in oxidatively modified plasma proteins and TNF-α levels. Linear mixed-effects models will be fitted where each patient will be entered into the model as a random effect. Fixed effect indicator variables will also be included for the sequence (mesna first, saline next or vice-versa), the period (first or second), the treatment effect (mesna vs. saline) and whether the tumor type is lymphoma or breast. In exploratory fashion, the interaction between tumor type and treatment sequence will be assessed although this study may be underpowered to adequately assess such an interaction. Secondary endpoints will investigate reduction in markers of cardiac stress (BMP and troponinI) and will also be assessed using linear mixed models.

12.2 Sample Size/Accrual Rate

Pilot data (pre and post measurements of plasma HNE) were collected on 5 patients administered mesna and 7 patients not administered mesna. Post minus pre mean differences were -34 in the mesna group and 38 in the comparison group (pooled std = 35.1). With a two-sided t-test setting alpha=0.05 and a power of 90%, one would need to enroll 6 patients per group to detect this large of an effect size (72/35.1).

We propose to take a conservative approach in powering the proposed study by dampening theobserved differences in means (72) from our pilot data by a factor of 1/2 (while assuming the samepooled standard deviation of 35.1). Therefore, we propose to enroll and collect pre and post plasma HNE values on 32 patients total (16 in each group), which will result in having 80% power (atalpha=0.05) to detect a between group difference of 36 in the mesna vs. the saline control groups.

We therefore plan to recruit 40 participants in two years to account for attrition.The study isconservatively powered to assess differences in oxidative modification of plasmaproteins to account for possible participant drop-out. However, even if the study has excellent retention the added sample size should permit a more thorough exploratory investigation of our secondary objectives.

Sample size = 40
Expected accrual rate = 2 / month

12.3 Stratification Factors

Patients will be randomized using stratified block randomization with stratification for tumor type between lymphoma and breast cancer. Patients will undergo stratified block randomization to be assigned to receive mesna during cycle 1 or 2 of therapy. The participant will receive a saline control during the other cycle not receiving mesna.

12.4 Analysis of Secondary Endpoints

cTnI and BNP will be measured to assess for markers of cardiac stress and oxidative proteins as well as neuronal damage markers for correlative studies will be analyzed using linear mixed models for each individual marker. Appropriate step-down tests may be conducted to assess differences at specific points in time during treatment sequence. Appropriate adjustments will be made for multiple comparisons (Tuckey and/or Scheffe adjustments). Should cTnI or BNP be non-normally distributed, analysis on either transformations of or the ranks of these data will be performed using the same linear mixed model framework. In all modeling paradigms, a test of the interaction between tumor type (breast or lymphoma) and treatment sequence will be conducted in an exploratory fashion as the study is likely underpowered to assess such interactions unless it is quite prominent.

REFERENCES

1. Kannarkat G, Lasher EE, Schiff D. Neurologic complications of chemotherapy agents. Current opinion in neurology 2007;20: 719-25.
2. Joshi G, Hardas S, Sultana R, St Clair DK, Vore M, Butterfield DA. Glutathione elevation by gamma-glutamyl cysteine ethyl ester as a potential therapeutic strategy for preventing oxidative stress in brain mediated by in vivo administration of adriamycin: Implication for chemobrain. J Neurosci Res 2007;85: 497-503.
3. Tangpong J, Cole MP, Sultana R, *et al.* Adriamycin-mediated nitration of manganese superoxide dismutase in the central nervous system: insight into the mechanism of chemobrain. Journal of neurochemistry 2007;100: 191-201.
4. Tangpong J, Cole MP, Sultana R, *et al.* Adriamycin-induced, TNF-alpha-mediated central nervous system toxicity. Neurobiology of disease 2006;23: 127-39.
5. Tangpong J, Sompol P, Vore M, St Clair W, Butterfield DA, St Clair DK. Tumor necrosis factor alpha-mediated nitric oxide production enhances manganese superoxide dismutase nitration and mitochondrial dysfunction in primary neurons: an insight into the role of glial cells. Neuroscience 2008;151: 622-9.
6. Andrykowski MA, Schmitt FA, Gregg ME, Brady MJ, Lamb DG, Henslee-Downey PJ. Neuropsychologic impairment in adult bone marrow transplant candidates. Cancer 1992;70: 2288-97.
7. Schagen SB, van Dam FS, Muller MJ, Boogerd W, Lindeboom J, Bruning PF. Cognitive deficits after postoperative adjuvant chemotherapy for breast carcinoma. Cancer 1999;85: 640-50.
8. Donovan KA, Jacobsen PB, Andrykowski MA, *et al.* Course of fatigue in women receiving chemotherapy and/or radiotherapy for early stage breast cancer. Journal of pain and symptom management 2004;28: 373-80.
9. Wefel JS, Lenzi R, Theriault R, Buzdar AU, Cruickshank S, Meyers CA. 'Chemobrain' in breast carcinoma?: a prologue. Cancer 2004;101: 466-75.
10. Wefel JS, Lenzi R, Theriault RL, Davis RN, Meyers CA. The cognitive sequelae of standard-dose adjuvant chemotherapy in women with breast carcinoma: results of a prospective, randomized, longitudinal trial. Cancer 2004;100: 2292-9.
11. Nelson HE. The National Adult Reading Test (NART): test manual. Windsor, Berks, UK: NFER-Nelson; 1982.
12. Delis DC, Kramer JH, Kaplan E. California Verbal Learning Test: adult version. San Antonio, TX: Psychological Corporation; 1987.
13. Wechsler D. WAIS-IV administration and scoring manual. San Antonio, TX: Psychological Corporation; 2008.
14. Wechsler D. Wechsler Memory Scale-III manual. San Antonio, TX: Psychological Corporation; 1997.
15. Reitan RM, Wolfson D. The Halstead-Reitan Neuropsychological Test Battery; theory and interpretation. Tucson, AZ: Neuropsychology Press; 1993.
16. Reitan RM, Wolfson D. The Haltead-Reitan neuropsychological test battery: theory and cllinical interpretation. Tucson, AZ: Neuropsychology Press; 1985.
17. Klove H. Clinical neuropsychology. Medical Clinics of North America 1963;47: 1647-58.
18. Benton AL, Hamsher K. Multilingual aphasia examination. Iowa City, IA: AJA Associates; 1989.
19. Spitzer RL, Kroenke K, Williams JB. Validation and utility of a self-report version of

PRIME-MD: the PHQ primary care study. Primary Care Evaluation of Mental Disorders. Patient Health Questionnaire. Jama 1999;282: 1737-44.
20. Hann DM, Jacobsen PB, Azzarello LM, *et al.* Measurement of fatigue in cancer patients: development and validation of the Fatigue Symptom Inventory. Qual Life Res 1998;7: 301-10.
21. Germanakis I, Anagnostatou N, Kalmanti M. Troponins and natriuretic peptides in the monitoring of anthracycline cardiotoxicity. Pediatric blood & cancer 2008;51: 327-33.
22. Lipshultz SE, Rifai N, Sallan SE, *et al.* Predictive value of cardiac troponin T in pediatric patients at risk for myocardial injury. Circulation 1997;96: 2641-8.
23. Soker M, Kervancioglu M. Plasma concentrations of NT-pro-BNP and cardiac troponin-I in relation to doxorubicin-induced cardiomyopathy and cardiac function in childhood malignancy. Saudi medical journal 2005;26: 1197-202.
24. Hayakawa H, Komada Y, Hirayama M, Hori H, Ito M, Sakurai M. Plasma levels of natriuretic peptides in relation to doxorubicin-induced cardiotoxicity and cardiac function in children with cancer. Medical and pediatric oncology 2001;37: 4-9.
25. Ganame J, Claus P, Eyskens B, *et al.* Acute cardiac functional and morphological changes after Anthracycline infusions in children. The American journal of cardiology 2007;99: 974-7.
26. Lipshultz SE, Sanders SP, Goorin AM, Krischer JP, Sallan SE, Colan SD. Monitoring for anthracycline cardiotoxicity. Pediatrics 1994;93: 433-7.
27. Steinherz LJ, Graham T, Hurwitz R, *et al.* Guidelines for monitoring of anthracycline cardiomyopathy: a rebuttal. Pediatrics 1994;94: 782-4.
28. Omenn GS. Exploring the human plasma proteome. Proteomics 2005;5: 3223, 5.
29. Aluise CD, St Clair D, Vore M, Butterfield DA. In vivo amelioration of adriamycin induced oxidative stress in plasma by gamma-glutamylcysteine ethyl ester (GCEE). Cancer letters 2009.

1. Declaration of Helsinki

WORLD MEDICAL ASSOCIATION DECLARATION OF HELSINKI
Adopted by the 18th World Medical Assembly
Helsinki, Finland, June 1964

and amended by the
29th World Medical Assembly, Tokyo, Japan, October 1975
35th World Medical Assembly, Venice, Italy, October 1983
41st World Medical Assembly, Hong Kong, September 1989
and the
48th General assembly,
Somerset West, Republic of South Africa, October 1996

INTRODUCTION

It is the mission of the physician to safeguard the health of the people. His or her knowledge and conscience are dedicated to the fulfillment of this mission.

The Declaration of Geneva of the World Medical Assembly binds the physician with the words, "The health of my patient will be my first consideration," and the International Code of Medical Ethics declares that, "A physician shall act only in the patient's interest when providing medical care which might have the effect of weakening the physical and mental condition of the patient."

The purpose of biomedical research involving human subjects must be to improve diagnostic, therapeutic and prophylactic procedures and the understanding of the etiology and pathogenesis of disease.

In current medical practice most diagnostic, therapeutic or prophylactic procedures involve hazards. This applies especially to biomedical research.

Medical progress is based on research which ultimately must rest in part on experimentation involving human subjects.

In the field of biomedical research a fundamental distinction must be recognized between medical research in which the aim is essentially diagnostic or therapeutic for a patient, and medical research, the essential object of which is purely scientific and without implying direct diagnostic or therapeutic value to the person subjected to the research.

Special caution must be exercised in the conduct of research which may affect the environment, and the welfare of animals used for research must be respected.
Because it is essential that the results of laboratory experiments be applied to human beings to further scientific knowledge and to help suffering humanity, the World Medical Association has prepared the following recommendations as a guide to every physician in biomedical research involving human subjects. They should be kept under review in the future. It must be stressed that the standards as drafted are only a guide to physicians all over the world. Physicians are not relieved from criminal, civil and ethical responsibilities under the laws of their own countries.

I. BASIC PRINCIPLES

1. Biomedical research involving human subjects must conform to generally accepted scientific principles and should be based on adequately performed laboratory and animal experimentation and on a thorough knowledge of the scientific literature.

2. The design and performance of each experimental procedure involving human subjects should be clearly formulated in an experimental protocol which should be transmitted for consideration, comment and guidance to a specially appointed committee independent of the investigator and the sponsor provided that this independent committee is in conformity with the laws and regulations of the country in which the research experiment is performed.

3. Biomedical research involving human subjects should be conducted only by scientifically qualified persons and under the supervision of a clinically competent medical person. The responsibility for the human subject must always rest with a medically qualified person and never rest on the subject of the research, even though the subject has given his or her consent.

4. Biomedical research involving human subjects cannot legitimately be carried out unless the importance of the objective is in proportion to the inherent risk to the subject.

5. Every biomedical research project involving human subjects should be preceded by careful assessment of predictable risks in comparison with foreseeable benefits to the subject or to others. Concern for the interests of the subject must always prevail over the interests of science and society.

6. The right of the research subject to safeguard his or her integrity must always be respected. Every precaution should be taken to respect the privacy of the subject and to minimize the impact of the study on the subject's physical and mental integrity and on the personality of the subject.

7. Physicians should abstain from engaging in research projects involving human subjects unless they are satisfied that the hazards involved are believed to be predictable. Physicians should cease any investigation if the hazards are found to outweigh the potential benefits.

8. In publication of the results of his or her research, the physician is obliged to preserve the accuracy of the results. Reports of experimentation not in accordance with the principles laid down in this Declaration should not be accepted for publication.

9. In any research on human beings, each potential subject must be adequately informed of the aims, methods, anticipated benefits and potential hazards of the study and the discomfort it may entail. He or she should be informed that he or she is a liberty to abstain from participation in the study and that he or she is free to withdraw his or her consent to participation at any time. The physician should then obtain the subject's freely-given informed consent, preferably in writing.

10. When obtaining informed consent for the research project the physician should be particularly cautious if the subject is in a dependent relationship to him or her or may consent under duress. In that case the informed consent should be obtained by a physician who is not engaged in the investigation and who is completely independent of this official relationship.

11. In case of legal incompetence, informed consent should be obtained from the legal guardian in accordance with national legislation. Where physical or mental incapacity makes it impossible to obtain informed consent, or when the subject is a minor, permission from the responsible relative replaces that of the subject in accordance with national legislation.

12. Whenever the minor child is in fact able to give consent, the minor's consent must be obtained in addition to the consent of the minor's legal guardian.

13. The research protocol should always contain a statement of the ethical considerations involved and should indicate that the principles enunciated in the present Declaration are complied with.

II. MEDICAL RESEARCH COMBINED WITH CLINICAL CARE
(CLINICAL RESEARCH)

1. In the treatment of the sick person, the physician must be free to use a new diagnostic and therapeutic measure, if in his or her judgment it offers hope of saving life, reestablishing health or alleviating suffering.

2. The potential benefits, hazards and discomfort of a new method should be weighed against the advantages of the best current diagnostic and therapeutic methods.

3. In any medical study, every patient - including those of a control group, if any--should be assured of the best proven diagnostic and therapeutic method.

4. The refusal of the patient to participate in a study must never interfere with the physician-patient relationship.

5. If the physician considers it essential not to obtain informed consent, the specific reasons for this proposal should be stated in the experimental protocol for transmission to the independent committee (I, 2).

6. The physician can combine medical research with professional care, the objective being the acquisition of new medical knowledge, only to the extent that medical research is justified by its potential diagnostic or therapeutic value for the patient.

III. NON-THERAPEUTIC BIOMEDICAL RESEARCH INVOLVING HUMAN SUBJECTS (NON-CLINICAL BIOMEDICAL RESEARCH)

1. In the purely scientific application of medical research carried out on a human being, it is the duty of the physician to remain the protector of the life and health of that person on whom biomedical research is being carried out.

2. The subjects should be volunteers--either healthy persons or patients for whom the experimental design is not related to the patient's illness.

3. The investigator or the investigating team should discontinue the research if in his/her or their judgment it may, if continued, be harmful to the individual.

4. In research on man, the interest of science and society should never take precedence over considerations related to the wellbeing of the subject.
