# Supplementary material for: Plasma TNF-α and Soluble TNF Receptor Levels after Doxorubicin with or without Co-Administration of Mesna—A Randomized, Cross-Over Clinical Study
Source: PLoS One. 2015 Apr 24;10(4):e0124988. doi: 10.1371/journal.pone.0124988 (PMC4409356; doi:10.1371/journal.pone.0124988)
Supplement: S1 Table — (DOCX) [file pone.0124988.s004.docx]

| **S1 Table.** Geometric Means and 95% Exponentiated CI’s for Log Levels for each biochemical marker by Randomization Groups | | | | | | | | |
| --- | --- | --- | --- | --- | --- | --- | --- | --- |
|  |  |  | Mesna:Saline (N=16) | | | Saline:Mesna (N=16) | | |
|  |  |  | Mean | 95% CI | | Mean | 95% CI | |
| **TNF Receptor 1** | Cycle 1 | Pre | 1145.96 | (898.21, | 1461.91) | 1465.28 | (1156.32, | 1856.97) |
|  |  | Post | 1103.01 | (861.00, | 1412.90) | 1484.75 | (1159.22, | 1901.50) |
|  | Cycle 2 | Pre | 1016.17 | (882.62, | 1169.93) | 1446.21 | (1114.65, | 1876.38) |
|  |  | Post* | 927.32 | (802.47, | 1071.70) | 1404.73 | (1094.55, | 1802.81) |
| **TNF Receptor 2** | Cycle 1 | Pre | 3447.48 | (2318.56, | 5126.10) | 4078.97 | (2833.59, | 5871.70) |
|  |  | Post | 2852.92 | (1889.94, | 4306.58) | 3754.09 | (2520.72, | 5591.48) |
|  | Cycle 2 | Pre | 2693.51 | (2275.83, | 3187.52) | 3804.73 | (2589.71, | 5589.81) |
|  |  | Post* | 2295.03 | (1918.69, | 2745.17) | 3292.16 | (2223.64, | 4873.66) |
| **TNF Alpha** | Cycle 1 | Pre | 3.68 | (1.98, | 6.81) | 3.55 | (1.96, | 6.42) |
|  |  | Post | 3.18 | (1.79, | 5.64) | 2.95 | (1.54, | 5.64) |
|  | Cycle 2 | Pre | 1.67 | (1.35, | 2.07) | 2.26 | (1.37, | 3.73) |
|  |  | Post* | 1.29 | (1.01, | 1.65) | 1.78 | (1.05, | 3.03) |
| **IL-18** | Cycle 1 | Pre | 474.28 | (315.48, | 712.94) | 428.85 | (261.36, | 703.73) |
|  |  | Post | 462.06 | (303.17, | 704.30) | 437.73 | (263.80, | 726.40) |
|  | Cycle 2 | Pre | 417.76 | (322.34, | 541.42) | 507.70 | (349.81, | 736.79) |
|  |  | Post* | 404.36 | (317.41, | 515.17) | 484.69 | (342.48, | 685.88) |
| **Protein Carbonyl** | Cycle 1 | Naïve Baseline | 76.13 | (62.21, | 93.17) | 80.74 | (67.21, | 96.99) |
|  |  | Post1/Pre1 | 73.54 | (48.09, | 112.42) | 108.82 | (88.20, | 134.26) |
|  | Cycle 2 | Pre2/Pre1 | 97.00 | (79.32, | 118.61) | 100.52 | (79.52, | 127.07) |
|  |  | Post2/Pre1* | 75.71 | (49.81, | 115.08) | 87.91 | (62.70, | 123.26) |
| **Plasma HNE** | Cycle 1 | Naïve Baseline | 0.76 | (0.68, | 0.84) | 0.74 | (0.65, | 0.83) |
|  |  | Post1/Pre1 | 102.63 | (94.74, | 111.17) | 98.95 | (89.06, | 109.94) |
|  | Cycle 2 | Pre2/Pre1 | 104.40 | (93.54, | 116.52) | 95.58 | (84.76, | 107.78) |
|  |  | Post2/Pre1* | 98.31 | (88.14, | 109.66) | 91.73 | (82.27, | 102.30) |
| **3NT** | Cycle 1 | Naïve Baseline | 0.95 | (0.87, | 1.03) | 0.89 | (0.78, | 1.02) |
|  |  | Post1/Pre1 | 108.75 | (101.98, | 115.99) | 104.28 | (99.57, | 109.20) |
|  | Cycle 2 | Pre2/Pre1 | 104.73 | (95.52, | 114.81) | 105.99 | (98.45, | 114.10) |
|  |  | Post2/Pre1* | 103.74 | (92.50, | 116.35) | 102.95 | (91.49, | 115.84) |
| **BNP** | Cycle 1 | Pre | 49.24 | (33.00, | 73.48) | 46.36 | (32.23, | 66.69) |
|  |  | Post | 47.17 | (31.30, | 71.07) | 50.73 | (34.16, | 75.35) |
|  | Cycle 2 | Pre | 46.30 | (31.15, | 68.82) | 45.85 | (36.19, | 58.08) |
|  |  | Post* | 46.66 | (32.03, | 67.97) | 54.33 | (41.72, | 70.73) |
| **Troponin** | Cycle 1 | Pre | 0.017 | (0.013, | 0.021) | 0.015 | (0.011, | 0.021) |
|  |  | Post | 0.012 | (0.010, | 0.015) | 0.014 | (0.010, | 0.019) |
|  | Cycle 2 | Pre | 0.016 | (0.012, | 0.021) | 0.020 | (0.014, | 0.029) |
|  |  | Post* | 0.019 | (0.014, | 0.026) | 0.021 | (0.014, | 0.030) |

* Timepoint occurs after crossover.
