# Supplementary material for: Plasma TNF-α and Soluble TNF Receptor Levels after Doxorubicin with or without Co-Administration of Mesna—A Randomized, Cross-Over Clinical Study
Source: PLoS One. 2015 Apr 24;10(4):e0124988. doi: 10.1371/journal.pone.0124988 (PMC4409356; doi:10.1371/journal.pone.0124988)
Supplement: S2 Table — (DOCX) [file pone.0124988.s005.docx]

| **S2 Table.** Parameter estimates from Repeated Measures Models for each measure. | | | | | |
| --- | --- | --- | --- | --- | --- |
|  |  | Estimate | Std Error | T statistic | p-value |
| **Model 1:**  **LOG**  **(TNF Receptor 1)** | Intercept | -0.375 | 0.665 | -0.56 | 0.578 |
|  | Treatment (Mesna vs. Saline) | 1.536 | 0.760 | 2.02 | 0.054 |
|  | Time (Pre2 vs. Post1) | 0.054 | 0.038 | 1.41 | 0.168 |
|  | AC vs. TA | -0.064 | 0.101 | -0.63 | 0.532 |
|  | CHOP vs. TA | -0.140 | 0.113 | -1.23 | 0.229 |
|  | Baseline Value | 1.062 | 0.094 | 11.31 | **<.0001** |
|  | Baseline Value* Treatment Interaction | -0.227 | 0.106 | -2.15 | **0.041** |
| **Model 2:**  **LOG**  **(TNF Receptor 2)** | Intercept | -0.421 | 0.622 | -0.68 | 0.505 |
|  | Treatment (Mesna vs. Saline) | 2.497 | 0.737 | 3.39 | **0.002** |
|  | Time (Pre2 vs. Post1) | 0.022 | 0.077 | 0.28 | 0.778 |
|  | AC vs. TA | 0.003 | 0.136 | 0.02 | 0.983 |
|  | CHOP vs. TA | -0.076 | 0.149 | -0.51 | 0.612 |
|  | Baseline Value | 1.045 | 0.077 | 13.50 | **<.0001** |
|  | Baseline Value* Treatment Interaction | -0.322 | 0.089 | -3.62 | **0.001** |
| **Model 3:**  **LOG (TNF Alpha)** | Intercept | -0.272 | 0.309 | -0.88 | 0.387 |
|  | Treatment (Mesna vs. Saline) | 0.275 | 0.208 | 1.32 | 0.197 |
|  | Time (Pre2 vs. Post1) | 0.389 | 0.162 | 2.41 | **0.023** |
|  | AC vs. TA | 0.012 | 0.334 | 0.04 | 0.972 |
|  | CHOP vs. TA | -0.204 | 0.369 | -0.55 | 0.585 |
|  | Baseline Value | 0.872 | 0.105 | 8.28 | **<.0001** |
|  | Baseline Value* Treatment Interaction | -0.315 | 0.120 | -2.62 | **0.014** |
| **Model 4:**  **LOG (IL-18)** | Intercept | 1.758 | 0.412 | 4.26 | **0.000** |
|  | Treatment (Mesna vs. Saline) | -0.148 | 0.085 | -1.74 | 0.094 |
|  | Time (Pre2 vs. Post1) | -0.024 | 0.114 | -0.21 | 0.836 |
|  | AC vs. TA | -0.019 | 0.185 | -0.10 | 0.917 |
|  | CHOP vs. TA | 0.053 | 0.194 | 0.27 | 0.786 |
|  | Baseline Value | 0.724 | 0.068 | 10.67 | **<.0001** |
| **Model 5:**  **LOG**  **(Protein Carbonyl)** | Intercept | 0.211 | 0.317 | 0.67 | 0.511 |
|  | Treatment (Mesna vs. Saline) | -0.201 | 0.152 | -1.33 | 0.195 |
|  | Time (Pre2 vs. Post1) | -0.099 | 0.115 | -0.86 | 0.397 |
|  | AC vs. TA | -0.005 | 0.329 | -0.01 | 0.989 |
|  | CHOP vs. TA | -0.204 | 0.318 | -0.64 | 0.526 |
| **Model 6:**  **LOG**  **(Plasma HNE)** | Intercept | -0.271 | 0.118 | -2.30 | **0.029** |
|  | Treatment (Mesna vs. Saline) | 0.062 | 0.057 | 1.08 | 0.288 |
|  | Time (Pre2 vs. Post1) | 0.009 | 0.036 | 0.24 | 0.810 |
|  | AC vs. TA | 0.247 | 0.123 | 2.00 | 0.055 |
|  | CHOP vs. TA | 0.261 | 0.119 | 2.19 | **0.037** |
| **Model 7:**  **LOG (3NT)** | Intercept | 0.062 | 0.085 | 0.73 | 0.470 |
|  | Treatment (Mesna vs. Saline) | 0.015 | 0.041 | 0.36 | 0.720 |
|  | Time (Pre2 vs. Post1) | 0.011 | 0.026 | 0.42 | 0.680 |
|  | AC vs. TA | -0.021 | 0.089 | -0.24 | 0.815 |
|  | CHOP vs. TA | -0.018 | 0.086 | -0.21 | 0.839 |
| **Model 8:**  **LOG (BNP)** | Intercept | 1.043 | 0.401 | 2.60 | **0.015** |
|  | Treatment (Mesna vs. Saline) | -0.072 | 0.1018 | -0.70 | 0.487 |
|  | Time (Pre2 vs. Post1) | 0.060 | 0.1354 | 0.44 | 0.661 |
|  | AC vs. TA | 0.127 | 0.229 | 0.56 | 0.583 |
|  | CHOP vs. TA | 0.091 | 0.217 | 0.42 | 0.679 |
|  | Baseline Value | 0.705 | 0.076 | 9.24 | **<.0001** |
| **Model 9:**  **LOG (Troponin)** | Intercept | -1.700 | 0.830 | -2.05 | 0.052 |
|  | Treatment (Mesna vs. Saline) | -0.225 | 0.147 | -1.53 | 0.139 |
|  | Time (Pre2 vs. Post1) | -0.345 | 0.113 | -3.06 | **0.005** |
|  | AC vs. TA | -0.157 | 0.306 | -0.51 | 0.614 |
|  | CHOP vs. TA | -0.231 | 0.311 | -0.74 | 0.465 |
|  | Baseline Value | 0.482 | 0.168 | 2.86 | **0.009** |
