# Supplementary material for: Plasma TNF-α and Soluble TNF Receptor Levels after Doxorubicin with or without Co-Administration of Mesna—A Randomized, Cross-Over Clinical Study
Source: PLoS One. 2015 Apr 24;10(4):e0124988. doi: 10.1371/journal.pone.0124988 (PMC4409356; doi:10.1371/journal.pone.0124988)
Supplement: S3 Table — (DOCX) [file pone.0124988.s006.docx]

| **S3 Table.** Spearman Correlations between timepoints within the same measure | | | |
| --- | --- | --- | --- |
| Correlation  (p-value) | Logpost1 with logpre1 | Logpre2 with logpre1 | Logpre2 with logpost1 |
| Log TNF ALPHA | \| **0.94038** \| \| --- \| \| **(<.0001)** \| | \| **0.63490** \| \| --- \| \| **(<.0001)** \| | \| **0.60979** \| \| --- \| \| **(0.0003)** \| |
| Log TNF Receptor 1 | \| **0.97177** \| \| --- \| \| **(<.0001)** \| | \| **0.88526** \| \| --- \| \| **(<.0001)** \| | \| **0.90689** \| \| --- \| \| **(<.0001)** \| |
| Log TNF Receptor 2 | \| **0.98900** \| \| --- \| \| **(<.0001)** \| | \| **0.82588** \| \| --- \| \| **(<.0001)** \| | \| **0.83798** \| \| --- \| \| **(<.0001)** \| |
| Log IL-18 | \| **0.98497** \| \| --- \| \| **(<.0001)** \| | \| **0.71957** \| \| --- \| \| **(<.0001)** \| | \| **0.74487** \| \| --- \| \| **(<.0001)** \| |
| Log PC | \| 0.05059 \| \| --- \| \| (0.7834) \| | \| -0.23057 \| \| --- \| \| (0.2042) \| | \| **0.44098** \| \| --- \| \| **(0.0115)** \| |
| Log Plasma HNE | \| 0.11217 \| \| --- \| \| (0.5411) \| | \| -0.06048 \| \| --- \| \| (0.7423) \| | \| 0.31378 \| \| --- \| \| (0.0803) \| |
| Log 3NT | \| -0.02273 \| \| --- \| \| (0.9017) \| | \| 0.02126 \| \| --- \| \| (0.9081) \| | \| **0.44831** \| \| --- \| \| **(0.0101)** \| |
