# Supplementary material for: Plasma TNF-α and Soluble TNF Receptor Levels after Doxorubicin with or without Co-Administration of Mesna—A Randomized, Cross-Over Clinical Study
Source: PLoS One. 2015 Apr 24;10(4):e0124988. doi: 10.1371/journal.pone.0124988 (PMC4409356; doi:10.1371/journal.pone.0124988)
Supplement: S4 Table — (DOCX) [file pone.0124988.s007.docx]

| **S4 Table.** All toxicities split by treatment and cycle | | | | | | | | |
| --- | --- | --- | --- | --- | --- | --- | --- | --- |
|  | Mesna | | | | Saline | | | |
|  | Cycle 1 (N=16) | | Cycle 2 (N=16) | | Cycle 1 (N=16) | | Cycle 2 (N=16) | |
|  | N | % | N | % | N | % | N | % |
| Alanine aminotransferase increased | 4 | 25% | 0 | 0% | 2 | 13% | 1 | 6% |
| Alkaline phosphatase increased | 1 | 6% | 0 | 0% | 1 | 6% | 0 | 0% |
| Allergic rhinitis | 1 | 6% | 0 | 0% | 1 | 6% | 0 | 0% |
| Anemia | 3 | 19% | 6 | 38% | 7 | 44% | 8 | 50% |
| Anorexia | 0 | 0% | 0 | 0% | 0 | 0% | 1 | 6% |
| Arthralgia | 1 | 6% | 0 | 0% | 1 | 6% | 0 | 0% |
| Aspartate aminotransferase increased | 1 | 6% | 0 | 0% | 0 | 0% | 0 | 0% |
| Blood bilirubin increased | 1 | 6% | 0 | 0% | 0 | 0% | 1 | 6% |
| Blurred vision | 0 | 0% | 0 | 0% | 0 | 0% | 1 | 6% |
| Bullous dermatitis | 0 | 0% | 1 | 6% | 0 | 0% | 0 | 0% |
| Chills | 0 | 0% | 0 | 0% | 2 | 13% | 0 | 0% |
| Constipation | 0 | 0% | 1 | 6% | 1 | 6% | 3 | 19% |
| Cough | 1 | 6% | 0 | 0% | 2 | 13% | 1 | 6% |
| Creatinine increased | 0 | 0% | 1 | 6% | 1 | 6% | 0 | 0% |
| Depression | 0 | 0% | 0 | 0% | 1 | 6% | 0 | 0% |
| Dermatitis | 1 | 6% | 1 | 6% | 0 | 0% | 1 | 6% |
| Diarrhea | 1 | 6% | 1 | 6% | 1 | 6% | 0 | 0% |
| Dyspnea | 0 | 0% | 1 | 6% | 1 | 6% | 1 | 6% |
| Edema limbs | 0 | 0% | 1 | 6% | 1 | 6% | 1 | 6% |
| Esophageal Spasms | 0 | 0% | 0 | 0% | 1 | 6% | 0 | 0% |
| Fatigue | 5 | 31% | 3 | 19% | 5 | 31% | 4 | 25% |
| Febrile neutropenia | 0 | 0% | 0 | 0% | 1 | 6% | 1 | 6% |
| Fibrinogen decreased | 0 | 0% | 0 | 0% | 1 | 6% | 0 | 0% |
| Flank pain | 0 | 0% | 0 | 0% | 1 | 6% | 1 | 6% |
| Gastritis | 0 | 0% | 0 | 0% | 0 | 0% | 1 | 6% |
| Gastroparesis | 1 | 6% | 0 | 0% | 0 | 0% | 0 | 0% |
| Headache | 2 | 13% | 0 | 0% | 1 | 6% | 3 | 19% |
| Hemorrhoids | 0 | 0% | 0 | 0% | 1 | 6% | 0 | 0% |
| Hyperglycemia | 0 | 0% | 0 | 0% | 1 | 6% | 0 | 0% |
| Hypernatremia | 0 | 0% | 0 | 0% | 1 | 6% | 1 | 6% |
| Hypoalbuminemia | 1 | 6% | 3 | 19% | 3 | 19% | 0 | 0% |
| Hypocalcemia | 0 | 0% | 5 | 31% | 2 | 13% | 1 | 6% |
| Hypoglycemia | 0 | 0% | 1 | 6% | 0 | 0% | 0 | 0% |
| Hypokalemia | 4 | 25% | 3 | 19% | 2 | 13% | 1 | 6% |
| Hypomagnesemia | 0 | 0% | 0 | 0% | 1 | 6% | 0 | 0% |
| Hyponatremia | 2 | 13% | 1 | 6% | 2 | 13% | 2 | 13% |
| Hypotension | 0 | 0% | 0 | 0% | 1 | 6% | 0 | 0% |
| Infected Toe | 0 | 0% | 0 | 0% | 0 | 0% | 1 | 6% |
| Insomnia | 0 | 0% | 0 | 0% | 1 | 6% | 1 | 6% |
| Lip infection | 0 | 0% | 0 | 0% | 1 | 6% | 0 | 0% |
| Lung infection | 0 | 0% | 0 | 0% | 0 | 0% | 1 | 6% |
| Lymphocyte count decreased | 8 | 50% | 2 | 13% | 0 | 0% | 10 | 63% |
| Mucositis oral | 1 | 6% | 0 | 0% | 1 | 6% | 1 | 6% |
| Muscle weakness upper limb | 0 | 0% | 0 | 0% | 0 | 0% | 1 | 6% |
| Myalgia | 1 | 6% | 0 | 0% | 1 | 6% | 2 | 13% |
| Nausea | 6 | 38% | 6 | 38% | 7 | 44% | 6 | 38% |
| Neutrophil count decreased | 9 | 56% | 1 | 6% | 7 | 44% | 2 | 13% |
| Non-cardiac chest pain | 1 | 6% | 1 | 6% | 1 | 6% | 0 | 0% |
| Pain | 1 | 6% | 1 | 6% | 2 | 13% | 0 | 0% |
| Pain in extremity | 0 | 0% | 0 | 0% | 1 | 6% | 0 | 0% |
| Palpitations | 0 | 0% | 0 | 0% | 0 | 0% | 1 | 6% |
| Paresthesia | 4 | 25% | 1 | 6% | 3 | 19% | 1 | 6% |
| Peripheral sensory neuropathy | 0 | 0% | 1 | 6% | 0 | 0% | 0 | 0% |
| Platelet count decreased | 1 | 6% | 2 | 13% | 4 | 25% | 0 | 0% |
| Pruritus | 1 | 6% | 1 | 6% | 1 | 6% | 0 | 0% |
| Rash maculo-papular | 0 | 0% | 0 | 0% | 0 | 0% | 1 | 6% |
| Skin infection | 1 | 6% | 0 | 0% | 0 | 0% | 0 | 0% |
| Sore throat | 0 | 0% | 1 | 6% | 0 | 0% | 1 | 6% |
| Tooth infection | 1 | 6% | 0 | 0% | 0 | 0% | 0 | 0% |
| Tremor | 1 | 6% | 0 | 0% | 0 | 0% | 0 | 0% |
| Tumor pain | 0 | 0% | 0 | 0% | 1 | 6% | 0 | 0% |
| Urinary frequency | 0 | 0% | 0 | 0% | 0 | 0% | 1 | 6% |
| Vaginal infection | 0 | 0% | 0 | 0% | 1 | 6% | 0 | 0% |
| Vomiting | 1 | 6% | 0 | 0% | 2 | 13% | 1 | 6% |
| Weight loss | 0 | 0% | 1 | 6% | 0 | 0% | 0 | 0% |
| Wheezing | 0 | 0% | 0 | 0% | 1 | 6% | 0 | 0% |
| White blood cell decreased | 10 | 63% | 6 | 38% | 8 | 50% | 2 | 13% |
| Wound infection | 1 | 6% | 0 | 0% | 0 | 0% | 0 | 0% |
| Total | 78 |  | 53 |  | 89 |  | 67 |  |
